# What works and why in the identification and referral of adults with comorbid obesity in primary care: A realist review

**DOI:** 10.1111/obr.12979

**Published:** 2019-12-22

**Authors:** David N. Blane, Sara Macdonald, Catherine A. O'Donnell

**Affiliations:** ^1^ General Practice and Primary Care Institute of Health and Wellbeing, University of Glasgow Glasgow UK

**Keywords:** obesity, primary care, realist synthesis, weight management

## Abstract

Primary care practitioners (PCPs) are well placed to *identify* individuals with obesity and weight‐related comorbidities and to *refer* them to weight management services (WMS), but this does not often happen in practice. In this realist review, we searched six databases for intervention studies targeted at PCPs to improve the identification and referral of adults with comorbid obesity. Realist analysis was used to identify context‐mechanism‐outcome (CMO) configurations across 30 included papers (reporting on 27 studies). Most studies used multiple intervention strategies, categorised into: (a) training, (b) tools to improve identification, (c) tools to improve ease of referral, (d) audit/feedback, (e) working in networks/quality circles, and (f) other. The realist synthesis identified 12 mechanisms through which interventions work to improve identification and referral, including increasing knowledge about obesity and awareness of and confidence in WMS among practitioners, improved communication and trust between practitioners and WMS, and higher priority given to weight management among primary care teams. The theory of “candidacy” (a person's eligibility for medical attention and intervention) provided a robust explanatory framework but required refinement: (a) to take account of the different services (primary care and weight management) that patients must navigate to access support; and (b) to acknowledge the importance of wider contextual factors.

## 1 BACKGROUND

Obesity is a risk factor for several noncommunicable diseases (NCDs), is widely regarded as a chronic disease, and is a major public health concern globally.^1^, ^2^, ^3^ Optimal care of patients with obesity is necessarily broad and holistic,^4^ but for adults with weight‐related comorbidities such as diabetes or heart disease, international guidelines recommend that primary care practitioners (PCPs) opportunistically identify such patients and offer signposting or referral to multidisciplinary weight management support.^5^, ^6^ Such identification is a key first step to all other aspects of care, including referral to other services, as well as management of obesity and its complications within primary care. However, obesity remains under‐identified and under‐treated in primary care,^7^, ^8^ even when it coexists with other chronic conditions, and there is marked variation in referrals to weight management services (WMS), and a high attrition rate between referral and attendance.^9^, ^10^ It is this under‐identification and under‐referral that is the particular focus on the work reported here.

Two recent syntheses of qualitative research have offered possible explanations for the suboptimal engagement with weight management by PCPs.^11^, ^12^ These can be divided into: patient factors (lack of resources, loss of motivation and self‐respect, and lack of confidence in care options); practitioner factors (lack of familiarity with and confidence in obesity care options, fear of causing offence, and viewing obesity as a social issue, unless there were associated comorbidities); and health system factors (which can either empower or disempower patients and practitioners).

Two systematic reviews of interventions have also been conducted in this area. The first assessed the effectiveness of interventions to change the behaviour of health professionals and/or the organisation of care to promote weight reduction in adults with overweight and obesity, and identified six RCTs.^13^ It found evidence of a change in clinicians' behaviours after receiving an educational intervention (eg, increased recording of weight), but no statistically significant difference in patient weight between intervention and control groups.

The second focused on studies of screening and opportunistic interventions for obesity and found no trials examining the effectiveness of primary care screening to identify overweight or obesity in adults.^14^ An update conducted in 2016 again found no trials in this area.^15^

This suggests that, while we have some insights into what works once practitioners *have identified* patients, we have little evidence about how best to promote and support the initial act of identification and referral. The lack of trials assessing effectiveness in this area also points to the need to take a broader, more holistic view of the available research evidence, while still paying attention to the rigour of that evidence. Accordingly, we aimed to identify what works and why in the identification and referral of adults with comorbid obesity in primary care. To do this, we adopted a realist approach, combining a systematic approach to literature searching with a realist, theory‐driven, approach to evidence synthesis.

## 2 METHODS

### 2.1 Search strategy and selection criteria

This was a realist review conducted according to RAMESES standards,^16^ as described in our protocol paper.^17^ The search strategy was based on the Cochrane review search terms,^13^ but with two key amendments. First, search terms for study type (eg, RCT) were removed to ensure that a wider range of interventions and approaches were included. Second, the timeframe used and the databases searched were changed to widen the scope of the search. The full search strategy can be found in Supp Data S1 and is summarised in Table 1.

**Table 1:** Summary of search strategy

Search terms used	Based around three concepts: Obesity/weight loss; Primary care; and Practitioner behaviour change (range of terms including training, protocol, referral, feedback, computer, etc.)
Databases searched	Medline, CINAHL, EMBASE, PsychINFO, Web of Science, Science Direct
Timeframe	Year 2004 to April 2017
Inclusion criteria	Intervention studies targeting primary care practitioners to improve the identification and referral of adults with obesity
Exclusions	Children Non‐English language No exclusions were set based on study type

Inclusion and exclusion criteria were established for title, abstract, and full paper screening; this process was facilitated by using the web‐based systematic review software DistillerSR (Evidence Partners, Ottawa, Canada). Two reviewers were involved at each stage, with conflicts discussed by the team. D.B. reviewed all articles at each stage. The role of “second reviewer” was divided between S.M. and C.O.D., with each doing half of the articles.

**Figure 1:**
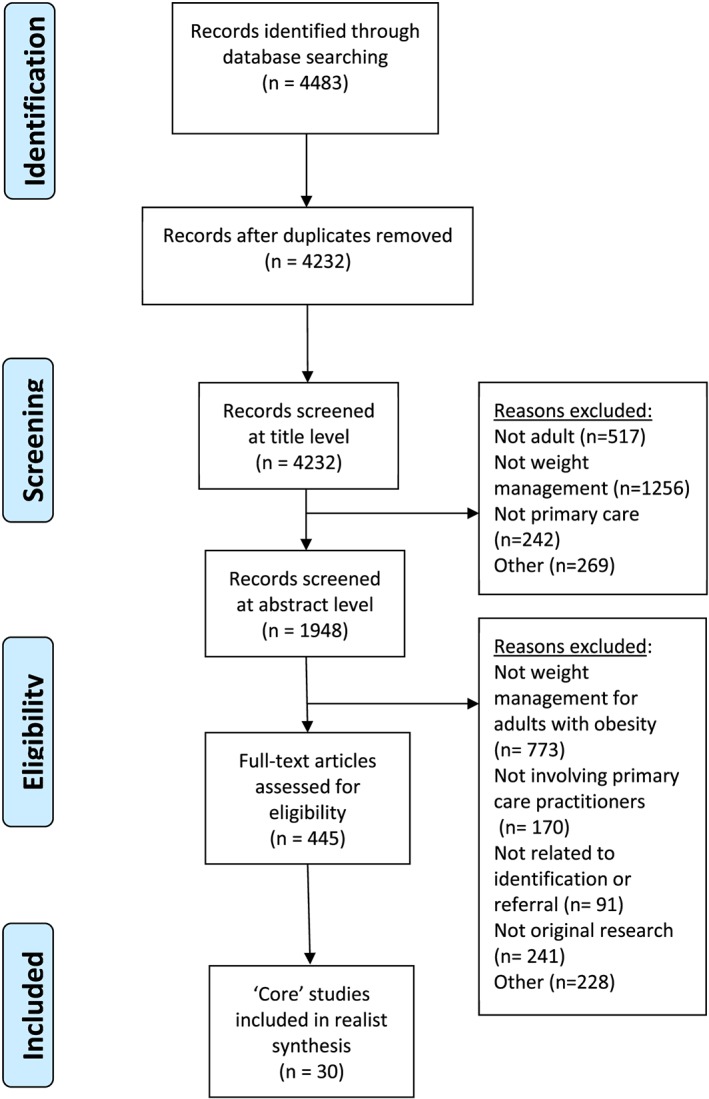
PRISMA flow diagram

The search of all six databases was conducted in May 2014 and updated in April 2017. In total, 4483 articles were retrieved. Removal of duplicates left 4232 articles for title screening. 1948 abstracts were screened, and 445 full text articles were assessed for eligibility. From these, 30 papers describing the most relevant intervention studies were included in the final synthesis. This process is presented in Figure 1 as a PRISMA flow diagram.^18^

### 2.2 Quality appraisal

The process of quality appraisal in a realist review is different to that from a traditional systematic review, with studies assessed principally on their relevance (to theory building and/or testing) and rigour (in terms of both reliability of methods and richness of description). However, a formal quality assessment was also carried out using a checklist for methodological quality of randomised and nonrandomised intervention studies.^19^ Studies were graded as “good,” “fair,” or “poor,” in terms of methodological rigour, based on their overall score. A score of >14 out of 23 was considered good, 10 to 14 was fair, and < 10 was poor. However, no study was excluded on the basis of methodological quality.

### 2.3 Descriptive analysis

A pre‐piloted proforma (Table S1) was used to extract data on study and participant characteristics as well as detailed information on the intervention, outcomes, context, and any suggestion of mechanisms.

In the first stage of analysis, each included study was broken down into its component parts, based on the intervention strategies reported (eg, tools, training, audit/feedback, or networks). Outcomes were charted for each study, including the three key outcomes of interest (discussion of weight, measuring and recording of weight and/or BMI, and referral to a WMS), as well as more proximal outcomes, such as markers of practitioner behaviour change (eg, self‐efficacy) or system‐level outcomes (eg, improved communication between WMS and practitioners) which could make the key outcomes more likely. Reference to underpinning theory was also recorde.

**Figure 2:**
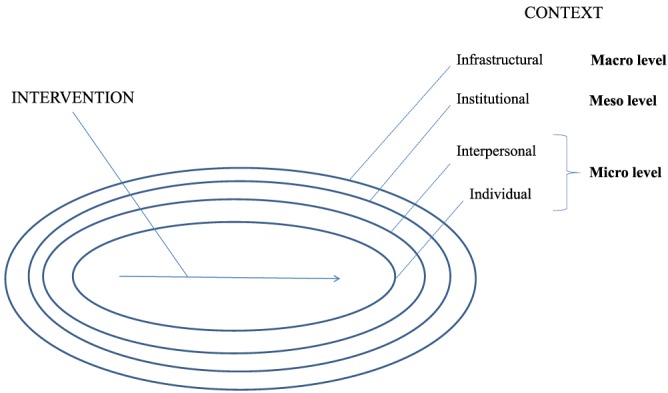
Levels of intervention context, adapted from Pawson^22^

### 2.4 Realist synthesis

Realist reviews may be considered, broadly speaking, as either theory building or theory testing.^20^ The current review investigates an under‐theorised area and is, therefore, more of the “theory‐building” type. Realist analysis sees reality as comprising multiple levels, which can be presented as micro, meso, and macro levels,^21^ or in Pawson's terms, individual, interpersonal, institutional, and infrastructural.^22^ Each level interacts with the others, providing important “contexts” in the “context‐mechanism‐outcome” (CMO) configuration, the heuristic device at the heart of realist analysis (Figure 2). See Table S2 for a glossary of realist terminology.

The second stage of analysis involved identifying CMO configurations within each study, a key step in the realist synthesis process. In keeping with previous realist reviews, we started with the three key outcomes of interest and “worked backwards” to discern potential mechanisms and contextual factors that affect those mechanisms.^23^, ^24^, ^25^ This process was facilitated by using “If‐Then‐Because” statements to represent (broadly) the elements of Context, Outcome, and Mechanism, respectively.^25^

These statements were developed as the review progressed and familiarity with data increased, but before formal data extraction was complete. As such, they should be viewed as a series of hypotheses, which could then be tested against empirical data from the included studies. This process was iterative, based on reflection on the potential mechanisms identified during data extraction, and through discussion with colleagues (supervisors and fellow realist researchers at the Centre for Advancement of Realist Evaluation and Syntheses at the University of Liverpool).

This began unpacking how contextual factors (operating at micro, meso, and macro levels), interacted with mechanisms to produce different outcomes. This process also identified “linked CMOs,” where completion of one CMO configuration led to a new CMO; for example, if identification of obesity was made possible because of prior recording.

The third stage involved exploring patterns within these CMO configurations. Potential mechanisms were compared across different studies and intervention strategies to assess if they were consistent in producing similar outcomes. For instance, would an electronic pop‐up reminding a practitioner to record BMI work through a similar mechanism as having a BMI chart on the consulting room wall?

The final stage of analysis involved configuring these CMO patterns into a coherent and plausible “refined” programme theory. As part of the process, several theoretical frameworks that could inform our data interpretation and synthesis were reviewed.^17^ From this, a “best fit” theory—candidacy theory—was identified and used to inform the final programme theory. Each stage of analysis was led by D.B. with discussion and agreement with S.M. and C.O.D. at regular meetings throughout the process.

## 3 RESULTS

### 3.1 Description of included papers

Our final sample consisted of 30 papers describing 27 studies. A detailed summary of the individual studies is in Table S3, where studies are described by author, location, study design, aim of the study, participants, and main outcome.

Most studies were from the USA (n = 23), with five from the UK and one each from Australia and Israel. Study designs included pre‐post (also known as before‐and‐after) studies (n = 11), quality improvement studies (n = 6), RCTs (n = 5), and nonrandomised controlled trials (n = 5). Ten studies were rated as “good,” nine as “fair,” and eleven as "poor.”

Although the focus of this review was on interventions targeting PCPs, few studies provided detailed information on practitioner characteristics, such as age^26^ and gender.^27^ Most of the practitioners involved were primary care/family medicine doctors, although six studies also included nurses or other allied health professionals.^27^, ^28^, ^29^, ^30^, ^31^, ^32^

As shown in Table S3, seven studies did not report any patient characteristics.^29^, ^33^, ^34^, ^35^, ^36^, ^37^, ^38^ Four studies reported on age and gender but did not provide any information on socio‐economic status (SES) or ethnicity.^39^, ^40^, ^41^, ^42^ The remaining studies were more likely to include ethnicity data than data on SES; when reported, studies used a proxy of individual SES such as education or insurance status, rather than a multidimensional marker of SES such as the Index of Multiple Deprivation (IMD).^43^

Only 12 contained any information about patient comorbidities.^26^, ^29^, ^30^, ^32^, ^39^, ^40^, ^41^, ^42^, ^44^, ^45^, ^46^, ^47^ Diabetes was recorded in all twelve of these, with hypertension in ten, CHD in nine, arthritis in six, and depression in five.

The total number of patients in all studies combined was 124 872, although more than half of this total (n = 85 472) came from just two studies.^28^, ^32^ The smallest study included just 87 patients.^48^ There were more females than males in every study that reported this data. The mean BMI was >30 kg/m^2^ in 15 of the 17 studies that reported this.

There were a range of outcomes measured in the 30 studies, although most included at least one of the key outcomes of interest to this review, namely:

- Discussion of weight (including lifestyle advice)^27^, ^28^, ^32^, ^33^, ^34^, ^36^, ^37^, ^38^, ^39^, ^40^, ^47^, ^49^, ^50^, ^51^, ^52^, ^53^, ^54^;
- Measuring and recording of weight and/or BMI^26^, ^27^, ^28^, ^29^, ^32^, ^36^, ^41^, ^42^, ^44^, ^47^, ^53^, ^55^; and
- Referral to WMS.^15^, ^26^, ^28^, ^29^, ^30^, ^31^, ^32^, ^36^, ^38^, ^41^, ^44^, ^45^, ^48^, ^51^, ^54^

Although weight loss was not a key outcome of interest in this review, changes in weight were reported in 11 of the included studies,^15^, ^30^, ^32^, ^40^, ^42^, ^45^, ^46^, ^47^, ^48^, ^50^, ^54^ and weight outcomes were made available on contacting the lead author of one further included study.^28^

### 3.2 Descriptive analysis by intervention type

Interventions were categorised according to the type of activity reported as follows:

1. Training
2. Tools/resources to improve identification of obesity
3. Tools/resources to improve ease of referral
4. Audit/feedback
5. Working in networks/Quality circles
6. Other strategies

Most of the studies were complex interventions, involving two or more intervention strategies and operating at different contextual levels. Table S4 provides more detail on each study according to intervention strategy, including information on the participants, intervention approach used, use of theory, and main outcomes reported.

1. Training

There was considerable heterogeneity across the studies, in terms of participants, training content, delivery and duration, use of theory, and outcomes measured. Most studies with training components involved primary care physicians,^33^, ^35^, ^38^, ^45^, ^47^, ^48^, ^52^ but one involved nurses.^40^ In terms of training content, most interventions aimed to increase participants' knowledge, skills, and attitudes related to obesity, usually involving identification/screening and brief intervention, including signposting or referral to other services. Two of the studies used the 5As framework of assess, advise, agree, assist, and arrange, while others incorporated guidelines for PCPs into their training content.

The delivery and duration of training varied markedly. Most studies involved group training sessions ranging from a few hours to several days, spread out over a period of months. Most described the theoretical underpinning of their training, whether related to the content (eg, 5As framework or motivational interviewing) or the approach (adult learning theory, organisational learning).

Most studies included at least one of the key outcomes of interest for this review, but it was not possible to determine the extent to which the outcomes presented were due to the training component per se, as most of the studies also involved additional intervention strategies. Three studies that only involved training^35^, ^48^, ^52^ reported increases in practitioner self‐efficacy to treat obesity,^35^ and improvements in the quality (though not the rate) of obesity counselling with an increase in referrals to weight management support.^48^, ^52^

- Tools/Resources to improve identification of obesity

There were eight studies in which tools or resources to improve the identification of obesity were the main intervention strategy and a further 10 studies where such tools were used in combination with other approaches.

The simplest tool was a laminated BMI chart.^55^ The study by Muo et al^41^ also involved BMI charts placed in consulting rooms, but in addition had a BMI chart reminder stamped into patients' notes. Several studies used charts posted above scales, in waiting rooms, in patient notes or on staff desks, acting as prompts for staff.^29^, ^40^, ^53^ Similarly, the relocation of scales to private locations within clinics and placement of working stadiometers conducive to work flow were found to facilitate BMI screening in the study by Erickson et al.^34^

Automatic BMI calculators integrated into the electronic medical record (EMR) featured in six studies.^26^, ^28^, ^31^, ^34^, ^42^, ^44^ Pop‐up reminders also featured, for example to recommend lifestyle modification for all adult patients with a BMI >25 kg/m^2^ 42 and electronic eligibility reminders based on age and BMI.^31^, ^39^

Several studies were more labour‐intensive, incorporating additional staff time. Examples included an electronic registry of patients with obesity (based on information collected during telephone counselling)^45^; the manual calculation of BMI by staff, which was then entered into the patient's EMR^26^; researchers manually adding obesity to the problem list^49^; or a member of staff (eg, nurse or rooming assistant) measuring a patient's height and weight prior to the medical consultation.^15^, ^27^

The most complex “tool” was a computer‐based intervention which involved the computer's expert system generating a “four‐ to five‐page individualised, tailored report that provided feedback addressing participant‐identified barriers to improving their physical activity and diet”.^50^

Few papers cited any formal theory related to the use of tools/resources to improve identification of obesity. However, most did cite supporting research evidence including the United States Preventive Services Task Force (USPSTF) guidelines^56^ and the 5As framework.^57^ Most of the studies reported positive outcomes, although some were mixed^27^, ^28^, ^29^, ^32^, ^41^, ^53^ and one showed no significant difference (in weight).^42^ Three studies only reported weight loss, with no information on rates of weight discussion, documenting of obesity, or referral.^42^, ^45^, ^50^

In the eight studies using tools alone to improve identification of adults with obesity, there were statistically significant increases in recording of BMI in patients' charts,^55^ increased documentation of obesity,^26^, ^41^, ^44^, ^53^ increased advice,^49^ and increased referral to other sources of support.^26^, ^44^ Similarly, in the remaining studies, there were statistically significant increases in recording of BMI in patient's charts,^29^ increased documentation of obesity,^28^ increased advice,^27^, ^39^, ^40^ and increased referral to other sources of support.^15^, ^31^, ^39^

- Tools/Resources to improve ease of referral

Four papers (from two intervention studies, Take Charge Lite [TCL]^30^, ^31^ and eLinkS^39^, ^54^) used tools and resources focused on improving referral; a further four incorporated tools as part of a wider intervention. TCL included BMI calculation and electronic reminders, as described above, but also the use of a single computer keystroke to print a TCL prescription that was accompanied by a letter describing the free weight management programme, with the telephone number to call to schedule an appointment. This resulted in an increase in referral from 5% at baseline to around 20%. In eLinkS, the EMR was again used as the platform for the intervention by making it fast and easy to refer patients to intensive counselling outside the office, but there was an additional focus on establishing bidirectional communication between practices and community weight loss counsellors, with participants given the choice of group classes offered through a commercial weight loss programme (Weight Watchers); individual telephone weight loss counselling; computer‐based counselling; or usual care. Although statistical differences were not reported, eLinkS also found an increase in the percentage of patients with obesity who received advice and referral.

The other four studies involved a database of community programmes and a health behaviour prescription pad,^51^ reminders with tailored management recommendations and a weight management screen including referral options,^28^ the provision of a complete list of local services and referral pathways,^32^ and an additional member of staff (from the research team) who ensured that patients who agreed to referral left the practice with an appointment.^15^ As with the other studies, outcomes from these four papers were generally positive, with the exception of the Goodfellow study, which found self‐reported increases in knowledge, confidence and skills related to weight management, but no significant differences in the proportion of patients offered a weight management programme.^32^

- Audit/feedback

The fourth intervention strategy was audit and feedback. There were seven studies that used audit and feedback as part of multicomponent interventions, although only one where it was the main strategy used.^33^ Different approaches were adopted, with some studies—for instance, the Counterweight study^40^, ^46^ and Schuster et al^47^—providing only a one‐off feedback of baseline performance related to current levels of obesity screening and intervention. The other studies provided repeated feedback, ranging in frequency from weekly, with an audit after 3 weeks^29^ to monthly audits^33^, ^38^, ^45^ to quarterly.^34^

The content of the feedback and person delivering it also varied; for instance, Ely et al used written feedback reports which included reminders of obesity care recommendations as well as patient‐specific information on barriers and facilitators to weight loss.^45^ In contrast, Aspy et al used practice enhancement assistants who worked closely with the practice team to modify office routines, forms, and computer templates, and help each team identify community resources.^33^

Use of theory was more prominent in these studies, including Plan‐do‐study‐act (PDSA) cycles^33^, ^38^ and the Theory of Planned Behaviour.^29^ Most of the included studies that used audit and feedback as an intervention strategy reported positive outcomes. These included increases in lifestyle interventions,^33^, ^40^ increased recording of obesity management,^29^, ^47^ improved adherence to obesity guidelines,^34^ and weight loss.^45^, ^46^

- Networks/Quality circles

Five papers (related to four studies) reported on the use of networks or quality circles. In the paper by Sinfield et al,^36^ a form of quality circle called a facilitated implementation group explored the use of tailoring to improve adherence to NICE guidelines on adult obesity in primary care. Tailoring involved two key steps. The first involved investigation of context and barriers to change; the second step involved the selection of intervention methods chosen to address the barriers identified. While this paper did not provide empirical evidence of improvements in identification and referral of adults with obesity, it provided invaluable insights into potentially supportive or constraining mechanisms involved, which resonated strongly with other findings from this review, presented in the next section.

Three other studies used slightly different approaches to quality circles. In the Counterweight study,^40^, ^46^ weight management advisers (all registered dietitians) provided regular peer support, once or twice each month, to practice nurses until they achieved competency and confidence in giving patients advice. This mentoring process usually took 6 months, and also contained elements of training and audit/feedback strategies.

In the study by Aspy and colleagues,^33^ a practice enhancement assistant met with the three clinician teams in each cluster and the principal investigator on three occasions (at 2, 4, and 6 months) to review progress and share ideas. These meetings were multidisciplinary, with clinicians, nurses or medical assistants, and office managers from each practice taking part. Finally, in the Combating Obesity at Community Health Centres (COACH) study,^38^ the quality circle (or Quality improvement collaborative) involved learning sessions, a website for evaluation, and conference calls for knowledge sharing.

In terms of use of theory, both the Aspy and Wilkes studies^33^, ^38^ used quality improvement tools such as PDSA cycles, while the Counterweight study^40^, ^46^ referred to learning theories and theories of innovation.

The studies that used quality circles generally reported positive outcomes, although most were multicomponent making it hard to discern which component(s) was most effective. As noted above, the study in which quality circles were the main strategy did not report outcomes related to identification and referral,^36^ but was kept in the review for its theoretical utility.

- Other interventions

Several studies used other intervention strategies over and above the five already outlined. Patient education/information materials were common, including body mass index brochures, patient action plan template, food/activity logs, portion control plates/handouts, home exercise routines, calorie counters, community resource brochures, and food and fat models.^28^, ^29^, ^32^, ^34^, ^37^, ^40^, ^42^, ^45^, ^46^, ^50^, ^51^

On the face of it, these resources may not obviously relate to improving practitioner identification and referral of adults with obesity; however, these resources helped to “minimize concerns regarding lack of time” for providers.^29^ This in turn may make providers feel more able to initiate a discussion around weight management. Similarly, the provision of a one‐page *Your Weight and Health Profile* form, recommended by the NIH,^58^ aimed “to enhance [practitioners'] ability to quickly assess readiness to lose weight,” which in turn could make referral more likely, or more appropriate.^29^

Incentives were cited in a few studies, including incentives to take part in training initiatives (eg, by providing Continuing Professional Development accreditation),^35^ incentives (eg, gift certificates) for referring the most patients,^38^ or the incentive of financial reimbursement for the diagnosis of obesity as a medical condition (in the USA).^53^

Two studies reported on the use of a designated lead responsible for implementation of the intervention in the practice.^32^, ^34^ Very little detail was provided on this leadership role in the Erickson paper,^34^ but the Goodfellow paper described the lead being well supported (monthly telephone calls), working closely with the research team to improve their knowledge and identifying additional resources and tools.^32^

Finally, two other strategies were used in one study each. These were the use of employee wellness initiatives or worksite wellness policies^34^ and the use of external accountability by implementing planned follow‐up.^15^

### 3.3 Realist synthesis

**Figure 3:**
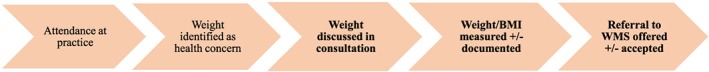
Initial “rough” programme theory

An early programme theory was developed based on clinical experience, familiarity with literature in this area, and a related qualitative study.^59^ Figure 3 depicts the process of identification and referral in five basic steps, although in practice the steps will not always follow sequentially, eg, measurement of weight may come before discussion of weight.

Steps 1 and 2 depend on patients attending their practice and then either them, or their PCP, identifying weight as an issue during the consultation. While important, these steps were not the main focus of this study, which was concerned with outcomes most amenable to practitioner behaviour change (steps 3 to 5).

The next stage in the synthesis involved developing a series of CMO configurations, informed by “If‐Then‐Because” statements (see Table S5).

### 3.4 Context‐mechanism‐outcome configurations by intervention strategy

Table 2 presents CMO configurations broken down by intervention strategy. The mechanisms have been presented here as “resources” plus “reasoning,” in keeping with the approach of Pawson and others,^22^, ^60^, ^61^ with a separate column for contexts (generally enabling but occasionally constraining).

**Table 2:** Context‐mechanism‐outcome configurations

**Intervention Strategy**	**Mechanism** Underlying Program Theory (Resources)		**Mechanism** Cognitive/Emotional Response (Reasoning)	**Potential Outcomes (+/ −)**
		Enabling/Constraining Contexts		
*Training*				
***Training***, eg, around brief interventions^15^, ^27^, ^29^, ^32^, ^33^, ^34^, ^35^, ^38^, ^40^, ^46^, ^47^, ^48^, ^50^, ^52^	Knowledge Skills Time/space for reflection	***ENABLING*** Supportive atmosphere Feedback provided Convenience of training setting Incentives to take part in training (eg, CPD points) ***CONSTRAINING*** Patients with a higher BMI were more likely to receive counselling	Increased **confidence** Increased self‐efficacy Increased **awareness** of referral options	Increased discussion of weight Increased referral rates
*Tools/resources to improve the identification of obesity*				
***Office‐based prompts*** Including desk‐based prompts such as flip‐charts^40^ and BMI charts in consulting rooms^29^, ^32^, ^55^; BMI chart and stamp in notes^,^ 41 posters on walls,^32^ and written handbook^37^	Physical reminder (practitioner) Knowledge of own BMI (patient)	***ENABLING*** Adequate time in consultation Repeated opportunities in primary care ***CONSTRAINING*** Physicians still had to manually calculate BMI—this needs to be automated^41^	**Opens safe space** for conversation More likely to think about BMI Objective measure **less stigmatising**	Increased discussion of weight Increased documentation of BMI and obesity Patient more likely to raise issue themselves, which makes practitioner more comfortable
***Automatic calculation of BMI in Electronic Medical Record*** 28, 30, 31, 44	Physical reminder to practitioner Memory, attention, and decision processes	***ENABLING*** Depends on patient BMI and may depend on patient comorbidities ***CONSTRAINING*** Danger of “alert fatigue”	More likely to think about BMI Objective measure **less stigmatising** (Doctors remain more influenced by patient appearance than by BMI)	Increased documentation of obesity Increased management of obesity
***Reminder card*** 53 ***or sticker placed on notes*** 47, 49 indicating diagnosis of obesity and recommending treatment/referral	Physical reminder to practitioner Memory, attention, and decision processes	***ENABLING*** Depends on patient BMI and may depend on patient comorbidities	More likely to think about BMI Increase in % physicians “comfortable” discussing obesity	Increased discussion of weight Increase in recording of obesity management in patient records
***Computerised support tool*** Tailored physician reports and patient self‐management goal sheet^50^; Automated clinical reminder for the clinician to recommend lifestyle modification for adults with obesity^34^, ^42^; Electronic registry of patients with obesity^45^	Self‐management goals selected prior to consultation. Included content on using motivational interviewing and other evidence‐based counselling styles.	***ENABLING*** **Physician support** and computer‐generated **tailored report** were more important to patients than the booklet, and some preferred to deal with these issues over several consultations.^50^ ***CONSTRAINING*** Time pressures and immediate health issues were barriers to addressing lifestyle change. Lack of services and long waiting lists were barriers to referral.	**Opened‐up space** for conversation—set stage. Prompts physician to consistently discuss lifestyle change **Increased practitioner confidence** and a reduction in their perception of barriers More likely to record obesity in patients who are actively working on losing weight.	Increased discussion of weight Increased documentation of obesity Increased referral to weight management resources
***Additional staff***, eg, “practice enhancement assistant”,^33^ “weight management advisers”,^46^ “clinic staff”,^26^, ^27^, ^39^ “health educator”^30^, ^31^; “research team”^15^	Identification of obesity made simpler by “additional” staff member routinely measuring height and weight Some worked closely with PCPs to modify routines, forms, computer templates	***CONSTRAINING*** Practitioners were more likely to drop the newly added screening items rather than drop the traditional physical measures.	Trust built up Additional time Social norms—make checking weight automatic, habitual	Increased identification of obesity Increased rate of brief interventions
*Tools/resources to improve the ease of referral*				
***Rapid referral***: ‐ TCL (Take Charge Lite) study ‐ “single computer keystroke” required to initiate referral^.^ 30, 31 ‐ eLINKS study—prompts and automated referrals^39^, ^54^ ‐ Weight management screen^28^	Ease of printing of TCL prescription Screen displays and EMR programming designed to make the interface with clinicians **easy and fast**, to automate the referral process electronically, and to facilitate proactive counselling. Patient choice was a factor here too.	***ENABLING*** Patient factors—reach highest for females, those aged 50 to 64, and non‐Hispanic Black patients. Increased awareness attributable at least in part to presentations, clinic brochures and posters, and feedback from participating patients. Convenience (of different services offered) and clinician recommendation were influencing factors.	Reminder for PCP to have further discussions re weight management with the patient. Increased Pt and PCP **awareness and acceptance** of the program Importance of co‐design (pre‐existing engagement) for trust in service	Increased discussion Increased referral
***Web‐based resource with database of community programs*** and patient education materials^51^	Improving links with community resources Leaflets, posters, adverts (eg, radio/paper) raising awareness of WMS	***CONSTRAINING*** Needs to be easily accessible	**Increased awareness** of available resources **Increased confidence**	Increased discussion of health behaviours Increased referral to WMS
***Improving links*** to community resources for weight management, eg, local service referral directory^30^, ^32^, ^38^	Improving links with community resources	***ENABLING*** Patient factors (older, female, higher BMI, comorbidities) Acceptance of referral depends on patient‐practitioner relationship and patient motivation	**Improved communication and trust** **Positive reinforcement** when positive results are seen	Increased discussion Increased referral
*Audit/feedback*				
***Feedback*** on individual or practice referral patterns^29^, ^33^, ^34^, ^38^, ^40^, ^45^, ^46^, ^47^	Social/group norms Benchmarking against other anonymized practices, regionally and nationally	***ENABLING*** Accuracy of data Time to discuss within practice Practices able to decide how much time to spend on different tasks	**Weight viewed as a priority** Peer comparison/competition may **spur on to improve practice** **Positive reinforcement** when positive results are seen	Increased discussion of weight Increased referral rates
*Working in networks/Quality circles*				
***Quality circles*** 33, 38; facilitated implementation groups^36^; peer support^40^, ^46^	Dedicated time Peer support Forming effective teams, setting aims, establishing measures, and spreading changes.	***ENABLING*** Participating health centres were given electronic data collection tools, and monthly data reports were required. Without such resources and financial support, it is unknown whether the Quality Improvement Collaboratives (QICs) could be implemented at community health centres	Increased knowledge, **confidence** and motivation **Consensus building** Increased trust among colleagues— “**safe space**” to discuss practice **Improved communication** within team	Increased discussion of weight Increased referral rates
*Other intervention strategies*				
***Incentives*** 35, 38, 53 ***Designated lead for weight management*** ^32,34^	Incentives for training (eg, CPD points) or for engagement with weight management (eg, gift certificates or financial reimbursement) Protected time and resource for lead practitioner	***ENABLING*** Support for lead is important ***CONSTRAINING*** Competing demands on time Depends on awareness and understanding of incentives	Practitioners **respond to financial or professional rewards** Weight is seen as a **priority** **Consensus** on management is built	Increased discussion of weight Increased recording of BMI and obesity diagnosis Increased referral

Recurring mechanisms (in “reasoning” column) are highlighted in bold

Breaking down the interventions to their CMO configurations highlighted the considerable repetition of mechanisms within many of these CMOs across intervention strategies. Examples included increased practitioner confidence in discussing weight, increased awareness of available services, and improved communication between primary care and WMS.

Comparing mechanisms across the interventions identified 12 through which, we propose, interventions targeted at PCPs to improve identification and referral of adults with obesity operate. Following the example of the realist review by Westhorp et al,^20^ each mechanism was labelled with a title, derived through discussion in the research team, which encapsulated how it worked. Table 3 presents the 12 mechanism titles according to the level at which they operate (individual, interpersonal, institutional), with a description and illustrative example for each.

**Table 3:** Mechanisms with illustrative examples

Level	Mechanism Title	Description	Illustrative Example from the Included Papers
**Individual**	*Yes we can*	PCPs have the confidence to talk about weight in a sensitive manner with their patients	*The GP has had a positive experience managing a patient with obesity leading to increased GP ‘professional self‐efficacy' to assist patients to change their behaviour. This has flowed into regular daily practice with the GPs reporting increased ease in discussing obesity and management options with patients who were not part of the pilot study*. (Sturgiss, 2017)
	*This matters*	PCPs recognise the importance and value of weight management	*The daily physical presence of a project director could increase communication about current guidelines for obesity, serve as a reminder to staff and providers that obesity is a clinic priority, and promote the adoption of new behaviours that are consistent with a focus on obesity as a health priority*. (Barnes, 2015)
	*Carrots and sticks*	PCPs respond to actual application of rewards/incentives or sanctions	*“Inclusion of weight management in GP contracts may be necessary however to promote wider involvement of primary care in managing obese patients.”* (Laws, 2004)
**Interpersonal**	*Right time, right place*	The consultation is considered a “safe space” to discuss weight	*Providers found patients using the BMI tables to calculate their own scores while waiting in the exam room for the providers. This information often prompted patients to begin a dialogue with the provider about the health implications of an elevated BMI, as well as possible strategies for weight loss*. (Lemay, 2004)
	*No blame, no shame*	The nonjudgmental, supportive approach taken by PCPs encourages engagement with weight management	*Providers reported that patient‐initiated discussions helped them feel more comfortable – and thus potentially more effective – discussing an objective measure, i.e. BMI, rather than a stigmatising label (overweight or obese) with their patients. In addition, the use of the objective measure, not one based on the providers' view of the patient's weight, helped decrease patients' defensiveness. (*Lemay, 2004)
**Institutional**	*It's working!*	Seeing positive outcomes operates as a positive feedback loop motivating further action	*That it takes a month or so of program implementation for reach to elevate likely reflects growing patient and PCP awareness and acceptance of the program attributable at least in part to presentations, clinic brochures and posters, and feedback from participating patients* (Clark, 2008)
	*Eyes and ears*	Current practice is monitored/audited and action is taken on the basis of this feedback	*At a wider practice level, provision of feedback data on practice performance was appreciated and provided an incentive to continue implementing the programme*. (Ross, 2008)
	*It's good to talk*	Improved communication between PCPs and weight management services results in increased trust and improved referrals	*During the monthly telephone calls and additional meeting, we assisted several practices to develop links with potentially useful local services, for example, an exercise class for people with limited mobility being run by a volunteer centre, or a health trainer service that offered one‐to‐one support in weight management*. (Goodfellow, 2016)
	*One size doesn't fit all*	A choice of weight management options is offered to patients in recognition of the heterogeneity of obesity	*Tailoring questions and advice to each site's unique situation, challenges, and priorities, made it possible to identify barriers and potential solutions to QI implementation quickly* (Wilkes, 2008)
	*Same hymn sheet*	Primary care teams are working well together, with consensus around weight management activities	*… because we've had [the Public Health Nurse] coming, checking in with us, and reminding us of things, and reviewing previously set goals, and assisting us in setting future goals . . . it's helped us stay on track, and it's helped us continue to be mindful of the process*. (Erickson, 2015)
	*Spread the word*	Awareness of available weight management services is raised among patients and practitioners	*Developing easily accessible mechanisms to link clinicians and patients to community resources could both raise awareness of these resources and facilitate guiding at‐risk patients to external resources for health behaviour change support*. (Flocke, 2006)
	*Quick and easy*	A key step in the process of identification and referral is automated to reduce practical barriers	*The screen displays and the EMR programming were designed to make the interface with clinicians easy and fast, to automate the referral process electronically, and to facilitate proactive counselling* (Krist, 2008)

[Abbreviations: GP, General practitioner/family doctor; PCP, primary care practitioner.

### 3.5 Contextual features influencing programme outcomes

The mechanisms identified in this review were affected by contextual factors operating at different levels (micro, meso, and macro). These contextual influences are outlined below and detailed with examples in Table S6.

#### 3.5.1 Microlevel contextual factors (individual/interpersonal)

The principal microlevel contextual factors that influenced outcomes were patient and practitioner characteristics, with patient BMI particularly important. The notion that PCPs are more likely to engage with weight for patients who are at the more severe end of the obesity spectrum, based on a visual assessment or judgement, featured in several studies.^41^, ^44^, ^49^ Similarly, several studies suggested that practitioners may be more likely to engage with weight as an issue when a patient has comorbidities.^30^, ^62^

Gender, age, ethnicity, and SES also influenced the likelihood of intervention success, with certain groups (especially middle‐aged women) more likely to engage with weight management than others.

Practitioner characteristics influencing outcomes were cited less often, but one study reported higher quality of obesity counselling from female practitioners and those who were more patient‐centred.^52^ This supports the idea that interpersonal, relational aspects of care are particularly important in obesity, which remains a highly stigmatised condition. The interpersonal context is most relevant to the mechanisms “No blame no shame” and “Right time right place.” Weight bias and stigma were cited in included studies as one of the barriers to practitioner engagement with weight (along with lack of time, lack of confidence, lack of training, and unwillingness to take responsibility^30^, ^33^, ^36^, ^40^, ^42^, ^47^, ^55^), but was only referred to in a handful of studies,^49^ reflecting a more general tendency of not considering or recording the unintended consequences of interventions.

#### 3.5.2 Mesolevel contextual factors (institutional)

Several institutional (meso) level factors that influenced outcomes were identified, reflecting the finding that most mechanisms operate at this level (Table 3). These factors included those related to the primary care consultation, the practice team, and the WMS to which PCPs were referring patients. At the level of the consultation, the issue of “alert fatigue” was raised by O'Grady and colleagues.^42^ This is when practitioners are faced with so many alerts and pop‐up reminders on their computers that they start to pay less attention to them, which may influence the success of automatic BMI calculators or similar EMR‐based prompts. The inflexibility of electronic medical record systems was identified, making adaptations difficult.^28^, ^33^

Second, with regard to the practice team, contextual factors that were highlighted as being likely to affect outcomes included staff turnover, practice culture and team working, and competing priorities. In a striking example of high staff turnover, participants in one study described the need for frequent orientation sessions to promote weight management programming to new providers.^38^

Creating a practice culture that routinely included a proactive approach to the diagnosis and treatment of obesity required significant leadership. One paper, reporting on the Provider and Health care team Adherence to Treatment Guidelines (PHAT‐G) intervention, described how its reliance on a part‐time project director may have affected the success of the project as opportunities for communication about the obesity guidelines, particularly face‐to‐face reminders, were limited.^29^

Another important finding was about the importance of the interdisciplinary team, with each role having its own responsibilities upon which other members of the team rely.^29^ National and international bodies also assert the importance of interprofessional teams in weight management,^63^ and several of the included studies and related papers endorsed this view.^29^, ^34^, ^64^

As for competing priorities, it was recognised that general practice is under pressure related to rising demands (ageing population with multimorbidity) and reduced workforce,^32^ meaning that weight management may not be seen as a priority.

A further mesolevel contextual factor related to the WMS themselves. As shown in many of the included interventions, strategies to improve links between primary care and local WMS often featured.^15^, ^28^, ^30^, ^31^, ^32^, ^39^, ^45^, ^51^, ^54^ Raising practitioner awareness of, and confidence in, a service was critical to the success of these interventions.^30^, ^31^ In the most striking example of contextual factors affecting the success of a WMS, the eLinks intervention was stopped after 5 weeks due to high demand using up the available funding.^39^ This example could equally be framed as a macrolevel issue of insufficient funding for weight management generally.

#### 3.5.3 Macrolevel contextual factors (infrastructural)

The final level at which contextual factors might influence intervention outcomes (by enabling or constraining the identified mechanisms) is the macro, or infrastructural, level. Three factors cited in the included studies will be considered: the normalisation of obesity as a result of its high prevalence; the timing of external events; and the funding (or lack of it) for weight management.

The normalisation of obesity was considered to have an impact on the outcome of identification of adults with obesity through its influence on both patients' and practitioners' perceptions of what a “normal” or healthy weight looks like.^41^ This resonates with other recent research, which found that the public's understanding of what a person with obesity looks like does not match the medical definition; perceptions of adults with obesity were of people who were much more overweight than the medical definition of obesity.^65^

The second macrolevel contextual factor that may have influenced outcomes was the timing of external events. This includes those that may have drawn energy away from implementing the intervention, such as other research collaborations,^51^ or changes in policy such as a new guideline, which could have minimised the observed effect of an intervention by influencing both intervention and control groups.^32^

The third and final macrolevel factor relates to the financing of a country's health system and funding for weight management. Most of the included studies were in the USA, a predominantly insurance‐based system, with copayments and significant gaps in health care coverage. This had implications for whether health care costs related to obesity would be reimbursed through health insurance. Obesity was officially recognised as a disease in the USA in 2011, when the Centre for Medicare and Medicaid services (CMS) announced that Medicare would cover intensive behavioural counselling for patients with obesity.^66^ This was reflected in the included studies from the USA, with those carried out prior to 2011 more likely to mention lack of reimbursement as a barrier.^26^ Practitioners are less likely to refer to a WMS—and patients are unlikely to attend—if the costs of that service are not covered by the patient's health insurance company.

### 3.6 Linking findings to middle‐range theory

The protocol paper described several theoretical frameworks—operating at different levels—which could be applied to this area of research.^17^

For the purposes of this review, the middle‐range theory of candidacy was chosen as one with the best explanatory potential for this synthesis. In brief, candidacy describes the process by which a person's eligibility for medical attention and intervention is jointly negotiated between individuals and health services, a process which is constantly being defined and redefined through interactions between individuals and professionals, and which operates in the context of conditions influenced by the wider socio‐cultural, political, and economic environment.^67^, ^68^

The strength of candidacy theory in this context is that it explicitly encompasses the two foci of the review—identification and referral. Furthermore, it is genuinely “middle range” in that it is not too abstract but produces explanations that are testable. Candidacy theory incorporates individual (patient and practitioner), interpersonal, institutional, and infrastructural factors.^67^, ^68^

Table 4 explains the seven candidacy constructs in relation to access to WMS, drawing on the findings from this review. It is worth reiterating here that, while the constructs are presented in an apparently linear fashion (for the sake of simplicity), the process is inherently dynamic and iterative.^69^

**Table 4:** Candidacy constructs explained in relation to WMS

Candidacy Construct	Explanation in Relation to Access to WMS
*Identification of candidacy*	This relates both to how individuals with obesity identify themselves as being candidates for a service, but also to how health professionals identify patients as being candidates for the WMS. In terms of the interventions described here and the mechanisms associated with those, approaches which facilitated and supported professionals to have conversations with patients (by increasing confidence or facilitating weight measurement) supported identification.
*Navigation of services*	This relates to navigation of the primary care system and of the WMS. Both have their challenges.
*Permeability of services*	This relates to how easy it is to access the service. Interventions that improved communication between practices and WMS are more likely to improve permeability.
*Appearing at services and asserting candidacy*	The act of turning up and representing oneself in an interaction with a health professional. As with identification, a PCP can also assert candidacy on behalf of a patient.
*Adjudication by professionals*	This typically relates to the decision‐making or judgment made by the health professional—(a) whether to discuss weight (if it has not been raised by the patient); (b) whether to offer referral. This depends first on being aware of what services are available and how to access them. Also depends on how likely the PCP thinks the patient is to benefit, or, indeed, attend the service. Assessment of motivation here and other competing demands on patient.
*Offer of/resistance to service*	How a PCP “sells” the WMS to the patient will influence their likelihood of: (a) accepting the referral; and (b) attending the service. This review found that the offer of referral is influenced by PCP's awareness of, and confidence in, the WMS.
*Operating conditions and local production of candidacy*	This incorporates factors that influence the candidacy process. This review identified factors at the micro (individual/interpersonal), meso (institutional) and macro (infrastructural) levels.

**Figure 4:**
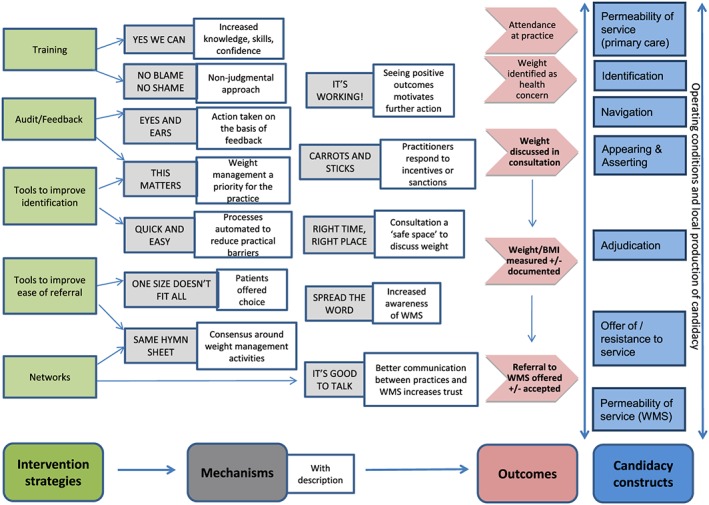
Linking intervention strategies, mechanisms, and outcomes with candidacy constructs

Figure 4 shows the links between the five main intervention strategies, the 12 mechanisms, outcomes (with three key outcomes of interest in bold), and the candidacy constructs. Each of the 12 mechanisms has a short description beside it. Most interventions operate through more than one mechanism.

As well as highlighting the links between the initial programme theory (Figure 3), we offer two modifications to the candidacy model first proposed by Dixon‐Woods et al^67^, ^68^ and modified by Mackenzie et al.^69^ First, we separate the “permeability of services” construct into two: permeability of primary care services and permeability of WMS, in recognition that both systems need to be navigated in order to achieve access to WMS. Second, we have expanded and moved the “Operating conditions and local production of candidacy” construct from the end of the process to a bidirectional arrow that spans the process. This is to reflect the finding from this review that contextual factors operate at different levels and can influence different steps in the candidacy process in different ways. For example, weight stigma or fear of causing offence may affect the likelihood of a PCP raising the issue of weight and identifying an adult with obesity.

## 4 DISCUSSION

### 4.1 Summary of findings and comparison with previous literature

The identification and management of obesity is a major issue for health care systems and practitioners internationally; this is the first realist review exploring adult weight management in primary care, with a focus on interventions to improve the identification and referral of adults with comorbid obesity. We identified and analysed 30 studies, from a total of 4232 papers screened. Most of the interventions described were complex interventions operating at multiple levels. The synthesis identified 12 mechanisms through which such interventions are proposed to work.

Some of these mechanisms operate at the individual level (“Yes we can,” “This matters,” and “Carrots and sticks”) and the interpersonal level (“Right time right place” and “No blame no shame”), but most operate at the institutional level, requiring changes to systems or culture at primary care practice level (“Quick and easy” and “Same hymn sheet”) or improved communication between practices and WMS (“Eyes and ears,” “It's good to talk,” “It's working!”, “Spread the word,” and “One size doesn't fit all”).

Importantly, this review also identified contextual factors, operating at different levels, which influence the extent to which these mechanisms are activated to produce desired outcomes. These include patient and practitioner characteristics, weight stigma and fear of causing offence, and competing priorities,

Finally, this synthesis has tested and further developed the theoretical concept of candidacy, identifying two key refinements. First, it is necessary to acknowledge that patients must present themselves to different services within a health system. This requires patients to continuously identify and present as “worthwhile” candidates to multiple services, with different sets of health care professionals. Second, we assert that the overarching importance of contextual factors was downplayed in the original candidacy framework. We believe that the “operating conditions” of candidacy act at multiple steps in the candidacy journey, and so need to be considered as an encompassing part of the framework. Acknowledging this extended role for contextual factors offers an opportunity to test this in a wider set of health‐related behaviours and practices.

Most of the included studies were complex and multifaceted, using combinations of the five main intervention approaches identified. However, it was not possible to identify which part of a complex intervention strategy contributed to the observed outcomes. The realist approach has aided the unpacking of these interventions into their component strategies and the identification of important contextual factors which can facilitate or hinder intervention success, thereby elucidating the “black box” of these complex interventions.^70^, ^71^

The improved effectiveness of combined interventions is supported by a recent theory‐led analysis of systematic reviews on the effectiveness of behaviour change interventions.^72^ The authors suggested that interventions which contribute to normative restructuring of practice, modify peer group norms and expectations (eg, educational outreach), and reinforce modified peer group norms by emphasising the expectations of an external reference group (eg, via reminders, or audit and feedback), offer the best chance of changing practitioner behaviour.^72^

The mechanisms identified in this review resonate with those described in two realist reviews exploring screening or referral by practitioners in other health care contexts.^73^, ^74^ In O'Campo et al's review of intimate partner violence screening across a range of health care settings,^74^ they also found that most studies were multicomponent. The four programme components that increased practitioner self‐efficacy for screening were institutional support, effective screening protocols, thorough initial and ongoing training, and immediate access/referrals to onsite and/or offsite support services.^74^ There are clear similarities between these four components and the effective intervention strategies used in the included studies in the present review. However, in that work, the authors were not able to draw out potential mechanisms that underpinned these strategies, or any of the enabling or constraining contextual factors.

In the realist review of physical health screening in people with mental health conditions by Lamontagne‐Godwin and colleagues,^73^ interventions were divided into those focusing on health service delivery changes (eg, staff training and protocol development) and those using tools designed to facilitate screening (eg, electronic prompts). As with the O'Campo study, the authors did not employ the CMO heuristic or make any attempt to discern mechanisms or theories of change underpinning the identified intervention strategies. They did, however, detail a range of barriers and facilitators to the successful implementation of both the health system delivery changes and the tools to facilitate screening. Some of the barriers resonate with those from this review, including resource constraints (eg, lack of time, staff turnover), environmental barriers (eg, poor communication between primary and secondary care), and unclear boundaries around professional role.^73^

This suggests that there is likely to be transferability of mechanisms involved in interventions to improve the identification and referral of patients in primary care across different clinical situations, in line with Pawson's thinking,^22^, ^75^ but further empirical testing of this assumption is required.

### 4.2 Strengths, limitations, and future research directions

The strengths of this review are, firstly, that it is the first realist review exploring the ways in which interventions designed to change practitioner identification and referral of patients with comorbid obesity operate in primary care. Second, the review adopted a comprehensive search strategy based on a previous Cochrane review but not restricted by study design, allowing for the incorporation of a broader body of relevant evidence. Third, it has not only used but substantially developed the theoretical framework of candidacy, making it more responsive to the impact that context has on both mechanisms and outcomes. Finally, the work has unpacked key interactions between theoretical mechanisms of intervention success and the enabling or constraining contexts in which these interventions take place, thus making an important potential contribution to policy and practice development in this area.

The main limitation of this review—in keeping with most realist reviews^76^—is that the primary data often lacked sufficient detail about the interventions, or their context, and were largely atheoretical, making it difficult to produce robust CMO configurations.^77^ Indeed, the 12 mechanisms proposed in this review and the range of contextual factors identified should be considered as preliminary and in need of further empirical testing. In particular, the focus on practitioner‐level interventions means that wider macrolevel factors were not so readily identifiable. Similarly, the included studies did not all give information on patient participants' obesity‐related comorbidities, or comment on the impact of those comorbidities on the processes of identification and referral. It is unlikely, however, that the findings would have been markedly different if studies had been excluded based on such details being lacking.

Finally, as well as theoretical literature, this review could have extended its search to include a wider range of empirical literature from different clinical settings (eg, smoking,^76^ alcohol,^78^ domestic violence^74^), which might have contributed to theory development.

With regard to future research directions, it would be of interest to test whether certain mechanisms from this review might apply to the four components (institutional support, effective screening protocols, thorough initial and ongoing training, and immediate access/referrals to onsite and/or offsite support services) identified in the O'Campo review: is it the sense of priority (eg, “This matters”) or the consistency of message (eg, “Same hymn sheet”) which is behind the importance of institutional support? Is it increased confidence (“Yes we can”) or improved awareness of available services (“Spread the word”) that make the links with other services work?

## 5 CONCLUSION AND RECOMMENDATIONS

Primary care practitioners are well placed to support adults with comorbid obesity, particularly by signposting or referring patients to WMS when appropriate. The findings from this review demonstrate the importance of good communication between WMS and primary care referrers to improve identification and referral processes. Successful interventions were usually multicomponent, including training of practitioners, audit/feedback on referrals, quality circles, and tools to aid both identification (eg, automatic BMI calculators, posters in waiting area) and referral. The mechanisms underlying successful strategies included increased knowledge about obesity and awareness of and confidence in WMS among practitioners, improved communication and trust between practitioners and WMS, and higher priority given to weight management among primary care teams.

Finally, we have not only confirmed that the middle‐range theory of candidacy has good explanatory potential in this area but have developed the model to more explicitly consider the contextual factors (at micro, meso, and macro levels) which influence candidacy. Further empirical testing of this model is recommended.

## FUNDING

This research was funded by the Scottish Government's Chief Scientist Office (CSO) as part of DB's ClinicalAcademic Fellowship (CAF 13/13).

## CONFLICT OF INTEREST

No conflict of interest was declared.

## ROLE OF THE FUNDING SOURCE

The funder of the study had no role in study design, data collection, data analysis, data interpretation, or writing of the report. The corresponding author had full access to all the data in the study and had final responsibility for the decision to submit for publication.

## COMPETING INTERESTS

All authors have completed the ICMJE uniform disclosure form at http://www.icmje.org/coi_disclosure.pdf and declare: no support from any organisation for the submitted work; no financial relationships with any organisations that might have an interest in the submitted work in the previous three years; no other relationships or activities that could appear to have influenced the submitted work.

## AUTHORS' CONTRIBUTIONS

D.B., C.O.D., and S.Mc.D. conceived the original idea, and collaborated at each stage of the research process, which was led by D.B. This paper is based on material first published in DB's PhD thesis. D.B. drafted the initial manuscript, and all other authors contributed to subsequent drafts. All authors read and approved the final manuscript.

## TRANSPARENCY DECLARATION

The lead author affirms that this manuscript is an honest, accurate, and transparent account of the study being reported; that no important aspects of the study have been omitted; and that any discrepancies from the study as planned (and, if relevant, registered) have been explained.

## DATA SHARING

Datasets currently held by lead author, D.B., and can be made available on request.

## Supporting information


**Table S1:** Data extraction form
**Table S2:** Realist terminology
**Table S3:** Detailed summary of included studies
**Table S4:** Studies broken down by intervention strategy
**Table S5:** If‐Then‐Because statements
**Table S6:** Contextual factors with illustrative examples
