# Supplementary material for: What works and why in the identification and referral of adults with comorbid obesity in primary care: A realist review
Source: Obes Rev. 2019 Dec 22;21(4):e12979. doi: 10.1111/obr.12979 (PMC7078935; doi:10.1111/obr.12979)
Supplement: Supplementary file 1 — Table S1: Data extraction form Table S2: Realist terminology Table S3: Detailed summary of included studies Table S4: Studies broken down by intervention strategy Table S5: If‐Then‐Because statements Table S6: Contextual factors with illustrative examples [file OBR-21-e12979-s001.pdf]

Manuscript ID OBR-08-19-4038 entitled "What works and why in the identification and referral of adults with comorbid obesity in primary care: a realist review"

David N Blane[1], Sara Macdonald[1], Catherine A O'Donnell[1*]

David N Blane, General Practice and Primary Care, Institute of Health and Wellbeing, University of Glasgow, 1 Horselethill Road, Glasgow, G12 9LX, UK.
David.blane@glasgow.ac.uk

Sara Macdonald, General Practice and Primary Care, Institute of Health and Wellbeing, University of Glasgow, 1 Horselethill Road, Glasgow, G12 9LX, UK.
Sara.macdonald@glasgow.ac.uk

Catherine A O'Donnell, General Practice and Primary Care, Institute of Health and Wellbeing, University of Glasgow, 1 Horselethill Road, Glasgow, G12 9LX, UK.
Kate.O'Donnell@glasgow.ac.uk

[*] Corresponding author: Catherine A O'Donnell
Email: Kate.O'Donnell@glasgow.ac.uk; Tel: 0141 330 8329

Supplementary Data S1: Full search strategy

Table S1: Data extraction form

Table S2: Realist terminology

Table S3: Detailed summary of included studies

Table S4: Studies broken down by intervention strategy

Table S5: If-Then-Because statements

Table S6: Contextual factors with illustrative examples

Supplementary Data S1: Full search strategy

OVID MEDLINE

1. exp Obesity/ or exp Obesity, Morbid/
2. (obes$ or overweight$).tw.
3. Weight Loss/
4. 1 or 2 or 3
5. exp Education, Continuing/
6. (education$ adj2 (program$ or intervention? or meeting? or session? or strateg$ or workshop? or visit?)).tw.
7. (behavio?r$ adj2 intervention?).tw.
8. *pamphlets/
9. (leaflet? or booklet? or poster or posters).tw.
10. ((written or printed or oral) adj information).tw.
11. (information$ adj2 campaign).tw.
12. (education$ adj1 (method? or material?)).tw.
13. outreach.tw.
14. ((opinion or education$ or influential) adj1 leader?).tw.
15. facilitator?.tw.
16. Practice Guideline as Topic/
17. *guideline adherence/
18. practice guideline?.tw.
19. (guideline? adj2 (introduc$ or issu$ or impact or effect? or disseminat$ or distribut$)).tw.
20. ((effect? or impact or evaluat$ or introduc$ or compar$) adj2 training program$).tw.
21. *reminder systems/
22. reminder?.tw.
23. (recall adj2 system$).tw.
24. (prompter? or prompting).tw.
25. *feedback/ or feedback.tw.
26. chart review$.tw.
27. ((effect? or impact or records or chart?) adj2 audit).tw.
28. compliance.tw.
29. marketing.tw.
30. or/5-29
31. exp Patient Care Team/
32. exp Primary Health Care/
33. exp Family Practice/ or exp General Practice/
34. exp *Health Personnel/
35. (team? adj2 (care or treatment or assessment or consultation)).tw.
36. (integrat$ adj2 (care or service?)).tw.
37. (care adj2 (coordinat$ or program$ or continuity)).tw.
38. (case adj1 management).tw.
39. *ambulatory care/
40. or/31-39
41. exp "Referral and Consultation"/
42. ((effect? or impact or evaluat$ or introduc$ or compar$) adj2 (treatment or care or screen$ or prevent$) adj2 program$).tw.
43. ((effect? or impact or introduc$) adj2 (legislation or regulations or policy)).tw.
44. *medical records/
45. *medical records systems, computerized/
46. (information adj2 (management or system?)).tw.
47. *utilization review/
48. *Quality Assurance, Health Care/
49. Quality of Health Care/
50. *program evaluation/
51. triage.tw.
52. *telephone/
53. (physician patient adj (interaction? or relationship?)).tw.
54. ((standard or usual or routine or regular or traditional or conventional or pattern) adj2 care).tw.
55. (program$ adj2 (reduc$ or increas$ or decreas$ or chang$ or improv$ or modify$ or monitor$ or care)).tw.
56. ((introduc$ or impact or effect? or implement$ or computer$) adj protocol?).tw.
57. (computer$ adj2 (diagnosis or decision?)).tw.
58. or/41-57
59. 30 or 58
60. 4 and 40 and 59

61. animal/
62. human/
63. 61 not (61 and 62)
64. 60 not 63
65. Child/
66. 64 not 65
67. limit 66 to (english language and yr="2004 -Current")

EMBASE (OVID)

1. exp Obesity/
2. (obes$ or overweight$).tw.
3. weight reduction/
4. or/1-3
5. exp medical education/
6. (education$ adj2 (program$ or intervention? or meeting? or session? or strateg$ or workshop? or visit?)).tw.
7. (behavio?r$ adj2 intervention?).tw.
8. publications/
9. medical information/
10. information dissemination/
11. information service/
12. (leaflet? or booklet? or poster or posters).tw.
13. ((written or printed or oral) adj information).tw.
14. (information$ adj2 campaign).tw.
15. (education$ adj1 (method? or material?)).tw.
16. outreach.tw.
17. ((opinion or education$ or influential) adj1 leader?).tw.
18. facilitator?.tw.
19. consensus conference?.tw.
20. exp Practice Guideline/
21. practice guideline?.tw.
22. (guideline? adj2 (introduc$ or issu$ or impact or effect? or disseminat$ or distribut$)).tw.
23. ((effect? or impact or evaluat$ or introduc$ or compar$) adj2 training program$).tw.
24. reminder system/
25. reminder?.tw.
26. decision support system/
27. (recall adj2 system$).tw.
28. (prompter? or prompting).tw.
29. *feedback/ or feedback.tw.
30. chart review$.tw.
31. ((effect? or impact or records or chart?) adj2 audit).tw.
32. compliance.tw.
33. marketing.tw.
34. or/5-33
35. patient care/
36. patient care planning/
37. general practice/
38. general practitioner/
39. nurse practitioner/
40. (team? adj2 (care or treatment or assessment or consultation)).tw.
41. (integrat$ adj2 (care or service?)).tw.
42. (care adj2 (coordinat$ or program$ or continuity)).tw.
43. (case adj1 management).tw.
44. case management/
45. exp primary healthcare/
46. *ambulatory care/
47. healthcare practice/
48. community health center/
49. healthcare facility/
50. *group practice/
51. medical practice/
52. or/35-51
53. *medical record/
54. (information adj2 (management or system?)).tw.
55. "peer review"/
56. "utilization review"/
57. clinical practice/

58. quality assurance.tw.
59. Outcome Assessment/
60. Total Quality Management/
61. Health Care Quality/
62. program evaluation/
63. triage.tw.
64. patient referral/
65. *telephone/
66. (physician patient adj (interaction? or relationship?)).tw.
67. *health maintenance organizations/
68. managed care.tw.
69. or/53-68
70. ((standard or usual or routine or regular or traditional or conventional or pattern) adj2 care).tw.
71. (program$ adj2 (reduc$ or increas$ or decreas$ or chang$ or improv$ or modify$ or monitor$ or care)).tw.
72. ((effect? or impact or evaluat$ or introduc$ or compar$) adj2 (treatment or care or screen$ or prevent$) adj2 program$).tw.
73. (computer$ adj2 (diagnosis or decision?)).tw.
74. ((introduc$ or impact or effect? or implement$ or computer$) adj protocol?).tw.
75. ((effect? or impact or introduc$) adj2 (legislation or regulations or policy)).tw.
76. or/70-75
77. 34 or 52 or 69
78. 4 and 77
79. nonhuman/
80. 78 not 79
81. 80
82. limit 81 to (english language and yr="2004 -Current")
83. child/
84. 82 not 83

<u>CINAHL (EBSCO)</u>

1. (MH “Obesity+”) or (MM “Weight Loss”)
2. TX (obes* or overweight*)
3. 1 or 2
4. (MH “Education, Continuing+”) or (MM “Pamphlets”) or (MM “Practice Guidelines”) or (MM “Professional Compliance”) or(MM “Reminder Systems”)
5. TX (education* N2 (program*or intervention* or meeting* or session* or strateg* or workshop* or visit*)
6. TX (behavior* N2 intervention*) or TI (behaviour* N2 intervention*) or AB (behavior* N2 intervention*)
7. TX (leaflet* or booklet* or poster or posters)
8. TX (written information) or TX (printed information) or TX (oral information)
9. TX (information* N2 campaign)
10. TX (education* N1 method*) or TX (education* N1 material*)
11. TX (outreach) or TX (facilitator*)
12. TX (opinion N1 leader*) or TX (education* N1 leader*) or TX (influential N1 leader)
13. TX (practice guideline*)
14. TX (guideline* N2 (introduc*or issu* or impact or effect* or disseminat* or distribut*))
15. TX ((effect* or impact or evaluat* or introduc* or compar*) N2 training program*)
16. TX (reminder*) or TX (recall N2 system*) or TX (prompter*) or TX (prompting)
17. TX (chart review*)
18. TX ((effect* or impact or records or chart*) N2 audit)
19. TX (compliance) or TX (marketing)
20. 4 or 5 or 6 or 7 or 8 or 9 or 10 or 11 or 12 or 13 or 14 or 15 or 16 or 17 or 18 or 19
21. (MH “Health Personnel+”)
22. (MH “Multidisciplinary Care Team+”) or (MM “Ambulatory Care”)
23. (MM “Family Practice”) OR (MM “Physicians, Family”)
24. TX (“patient care planning” or “case management”
25. TX (integrat* N2 care) or TX (integrat* N2 service*)
26. TX (care N2 (coordinat* or program* or continuity))
27. TX (chang* N2 location*) or TX (home N2 treat*)
28. 21 or 22 or 23 or 24 or 25 or 26 or 27
29. (MH “Medical Records+”)
30. (MM “Peer Review”) or (MM “Utilization Review”)
31. (MM “Quality Assurance”) or (MM “Outcome Assessment”) or (MM “Quality Improvement”) or (MM “Quality of Health Care”) or (MM “Program Evaluation”) or (MH “Referral and Consultation+”) or (MM “Patient History Taking”) or (MM “Telephone”)
32. (MM “Process Assessment (Health Care)”)

33. TX (computer* N2 diagnosis) or TX (computer* N2 decision*)
34. TX ((standard or usual or routine or regular or traditional or conventional or pattern) N2 care)
35. TX (program*N2 (reduc* or increas* or decreas* or chang* or improv* or modif* or monitor* or care))
36. TX ((effect* or impact or evaluat* or introduc* or compar*) N2 “treatment program*”)
37. TX ((effect* or impact or evaluat* or introduc* or compar*) N2 “care program*”)
38. TX ((effect* or impact or evaluat* or introduc* or compar*) N2 “screening program*”)
39. TX ((effect* or impact or evaluat* or introduc* or compar*) N2 “prevention program*”)
40. TX ((introduc* or impact or effect* or implement* or computer*) N2 protocol*)
41. TX (effect* N2 (legislation or regulations or policy)) or TX (impact* N2 (legislation or regulations or policy)) or TX (introduc* N2 (legislation or regulations or policy))
42. TX (information N2 management) or TX (information N2 system*)
43. TX (“physician practice patterns”) or TX (“quality assurance”)
44. TX (“triage” or “managed care”)
45. TX (“physician patient interaction*”) or TX (“physician patient relationship*)
46. 29 or 30 or 31 or 32 or 33 or 34 or 35 or 36 or 37 or 38 or 39 or 40 or 41 or 42 or 43 or 44 or 45
47. 20 or 46
48. 3 and 28 and 47
49. Restrictions to year 2004 onwards and English language

PsycINFO (EBSCO)
1. MJ Obesity or MJ Weight Loss
2. TX (obes* or overweight*)
3. 1 or 2
4. TX continuing education or TX physician education or TX Pamphlets or TX Practice Guidelines or TX Professional Compliance or TX Reminder Systems
5. TX (education* N2 (program*or intervention* or meeting* or session* or strateg* or workshop* or visit*)
6. TX (behavior* N2 intervention*) or TI (behaviour* N2 intervention*) or AB (behavior* N2 intervention*)
7. TX (leaflet* or booklet* or poster or posters)
8. TX (written information) or TX (printed information) or TX (oral information)
9. TX (information* N2 campaign)
10. TX (education* N1 method*) or TX (education* N1 material*)
11. TX (outreach) or TX (facilitator*)
12. TX (opinion N1 leader*) or TX (education* N1 leader*) or TX (influential N1 leader)
13. TX (practice guideline*)
14. TX (guideline* N2 (introduc*or issu* or impact or effect* or disseminat* or distribut*))
15. TX ((effect* or impact or evaluat* or introduc* or compar*) N2 training program*)
16. TX (reminder*) or TX (recall N2 system*) or TX (prompter*) or TX (prompting)
17. TX (chart review*)
18. TX ((effect* or impact or records or chart*) N2 audit)
19. TX (compliance) or TX (marketing)
20. 4 or 5 or 6 or 7 or 8 or 9 or 10 or 11 or 12 or 13 or 14 or 15 or 16 or 17 or 18 or 19
21. TX multidisciplinary practices OR TX ambulatory medical care
22. TX Health practitioners OR Health Personnel
23. TX family practice OR TX family physicians
24. TX (“patient care planning” or “case management”
25. TX (integrat* N2 care) or TX (integrat* N2 service*)
26. TX (care N2 (coordinat* or program* or continuity))
27. TX (chang* N2 location*) or TX (home N2 treat*)
28. 21 or 22 or 23 or 24 or 25 or 26 or 27
29. TX medical records
30. TX peer review OR TX utilization review
31. TX Quality Assurance OR TX Outcome Assessment OR TX Quality Improvement OR TX Quality of Health Care OR TX Program Evaluation OR TX Referral OR TX Telephone
32. TX Process Assessment
33. TX (computer* N2 diagnosis) or TX (computer* N2 decision*)
34. TX ((standard or usual or routine or regular or traditional or conventional or pattern) N2 care)
35. TX (program*N2 (reduc* or increas* or decreas* or chang* or improv* or modif* or monitor* or care))
36. TX ((effect* or impact or evaluat* or introduc* or compar*) N2 “treatment program*”)
37. TX ((effect* or impact or evaluat* or introduc* or compar*) N2 “care program*”)
38. TX ((effect* or impact or evaluat* or introduc* or compar*) N2 “screening program*”)
39. TX ((effect* or impact or evaluat* or introduc* or compar*) N2 “prevention program*”)
40. TX ((introduc* or impact or effect* or implement* or computer*) N2 protocol*)
41. TX (effect* N2 (legislation or regulations or policy)) or TX (impact* N2 (legislation or regulations or policy)) or TX (introduc* N2 (legislation or regulations or policy))
42. TX (information N2 management) or TX (information N2 system*)

43. TX (“physician practice patterns”) or TX (“quality assurance”)
44. TX (“triage” or “managed care”)
45. TX (“physician patient interaction*”) or TX (“physician patient relationship*)
46. 29 or 30 or 31 or 32 or 33 or 34 or 35 or 36 or 37 or 38 or 39 or 40 or 41 or 42 or 43 or 44 or 45
47. 20 or 46
48. 3 and 28 and 47
49. Restrictions to year 2004 onwards and English language

<u>**Web of Science**</u>

1. **TOPIC:** (obesity) *OR* **TOPIC:** (weight management) *OR* **TOPIC:** (weight loss)
2. **TOPIC:** (family practice) *OR* **TOPIC:** (general practice) *OR* **TOPIC:** (primary care) *OR* **TOPIC:** (care NEAR/2 (coordinat* OR program* OR continuity)) *OR* **TOPIC:** (health personnel)
3. **TOPIC:** (medical education) *OR* **TOPIC:** (education NEAR/2 (program* OR intervention OR meeting OR session OR strateg* OR workshop OR visit))
4. **TOPIC:** (publications) *OR* **TOPIC:** ((written OR printed OR oral) NEAR/2 information) *OR* **TOPIC:** (information NEAR/2 campaign) *OR* **TOPIC:** (education NEAR/2 (method OR material))
5. **TOPIC:** (outreach) *OR* **TOPIC:** ((opinion OR education* OR influential) NEAR/1 leader) *OR* **TOPIC:** (facilitator) *OR* **TOPIC:** (practice guideline) *OR* **TOPIC:** (reminder) *OR* **TOPIC:** (decision support system) *OR* **TOPIC:** (recall NEAR/2 system)
6. **TOPIC:** (guideline NEAR/2 (introduc* OR issu* OR impact OR effect* OR disseminat* OR distribut*))
7. **TOPIC:** ((effect* OR impact OR evaluat* OR introduc* OR compar*) NEAR/2 training)
8. **TOPIC:** ((effect* OR impact OR records OR chart*) NEAR/2 audit) *OR* **TOPIC:** (feedback) *OR* **TOPIC:** (compliance) *OR* **TOPIC:** (marketing) *OR* **TOPIC:** (recall NEAR/2 system*)
9. **TOPIC:** (((effect* OR impact OR evaluat* OR introduc* OR compar*) NEAR/2 (treatment OR care OR screen* OR prevent*) NEAR/2 program*))
10. **TOPIC:** (outcome assessment) *OR* **TOPIC:** (program evaluation) *OR* **TOPIC:** (triage) *OR* **TOPIC:** (referral) *OR* **TOPIC:** ((physician AND patient NEAR/2 (interaction OR relationship))) *OR* **TOPIC:** (managed care)
11. **TOPIC:** ((program* NEAR/2 (reduc* OR increas* OR decreas* OR chang* OR improv* OR modify* OR monitor* OR care)))
12. **TOPIC:** (((effect* OR impact OR introduc* OR implement* OR computer*) NEAR/2 (treatment OR care OR screen* OR prevent*) NEAR/2 protocol*))
13. **TOPIC:** (((effect* OR impact OR introduc*) NEAR/2 (legislation OR regulations OR policy)))
14. **#3 OR #4 OR #5 OR #6 OR #7 OR #8 OR #9 OR #10 OR #11 OR #12 OR #13**
15. **#1 AND #2 AND #14**

Refined by: **RESEARCH AREAS:** NUTRITION DIETETICS OR HEALTH CARE SCIENCES SERVICES OR COMMUNICATION OR PSYCHOLOGY OR SOCIAL SCIENCES OTHER TOPICS OR EDUCATION EDUCATIONAL RESEARCH OR BEHAVIORAL SCIENCES OR SOCIOLOGY OR COMPUTER SCIENCE OR ANTHROPOLOGY OR MEDICAL ETHICS OR RESEARCH EXPERIMENTAL MEDICINE OR PUBLIC ADMINISTRATION

Restricted to year 2004 onwards and English language.

<u>**Science Direct**</u>

(obesity OR weight loss) AND (primary care OR general practice OR family practice) AND (refer* OR education* OR screen* OR feedback OR training OR guideline OR evaluat* OR effect* OR identif*)

Decision Sciences
Medicine and Dentistry
Nursing and Health Professions
Psychology
Social Sciences

Article
Review article
Short survey

2004 to date

Limit to ‘topics’ **"patient, weight loss, bariatric surgery, primary care, health care, life style, diabetes"**

Table S1: Data extraction form

Data Extraction – done by (initials) on (date)

**Article Details**	
Study Title	
Authors	
Journal, Vol, Issues, Page nos.	

**Study Details**	**Provided**	**Not provided**	**Unclear**
Country study set in			
Reimbursement system (if known) e.g. fee-for-service, capitation, mixed?			
Year study conducted			
Setting of care (e.g. general practice, outpatient)			
**What is the research question or research objective(s)?**			
**How are participants sampled?** (e.g. theoretical, purposive, random)	Patients:<br><br>Practitioners:		
**How were participants recruited?**	Patients:<br><br>Practitioners:		
Participants – patients (e.g. adults with overweight/obesity/diabetes)?			
Mean BMI			
Number of patient participants			
Age of patient participants			
Gender			
Ethnicity			
Socio-economic status			
Co-morbidities mentioned (give details)			
Exclusions			
Participants – practitioners			

(e.g. GPs or PNs)?			
Level of training/Experience			
Proportion of eligible providers who participated			
Number of practitioners			
Age			
Gender			
Practice characteristics (e.g. urban, rural, singlehanded)?			
Exclusions			
What type of study is it? (e.g. RCT, cohort, qualitative etc)			
**Details of the intervention (e.g. what is the 'resource(s)' provided?)**			
Timing of the intervention (e.g. Frequency, duration, etc.)			
Intervention Recipient (individual or group)			
Intervention Deliverer (individual or group)			
Practitioner behaviour targeted? (e.g. increased referral)			
**Any suggestion by the authors of mechanisms of action of the chosen intervention strategies?**			
Consultation with recipients?			
**Evidence base of intervention? (e.g. any reference to 'theory' – either implementation or behaviour change or other?)**			
Barriers to change identified?			
Details of control conditions (if appropriate)			
Any indicators of acceptability to users?			
**RESULTS Primary outcome**			
Secondary outcome			
Losses to follow-up			
**Ethical approval sought and obtained?**			
How is data collected?			
How is data analysed? (e.g. grounded theory, thematic			

analysis)			
What is the overall conclusion or recommendations of the study?			
What (if any) study limitations are declared?			
How is the study funded? Are any conflicts of interest declared?			
**Reviewer's notes on 'what is going on here?' 'What are the mechanisms?'**			
**Other notes**			

**Reporting**	Yes (1)	No/Unclear (0)
1. *Is the **hypothesis/aim/objective** of the study clearly described?*		
2. *Are the **main outcomes** to be measured clearly described in the Introduction or Methods section?*		
3. *Are the **characteristics of the patients** included in the study clearly described?*		
4. *Are the **interventions of interest** clearly described?*		
5. *Are the **distributions of principal confounders** in each group of subjects to be compared clearly described?*		
6. *Are the **main findings** of the study clearly described?*		
8. *Have all important **adverse events** that may be a consequence of the intervention been reported?*		
9. *Have the characteristics of **patients lost to follow-up** been described?*		
**External validity**		
11. *Were the subjects **asked to participate** in the study representative of the entire population from which they were recruited?*		
12. *Were those subjects **who were prepared to participate** representative of the entire population from which they were recruited?*		

13. *Were the **staff, places, and facilities** where the patients were treated, representative of the treatment the majority of patients receive?*		
**Internal validity - bias**		
15. *Was an attempt made to **blind those measuring** the main outcomes of the intervention?*		
16. *If any of the results of the study were based on **"data dredging",** was this made clear?*		
17. *In trials and cohort studies, do the analyses **adjust for different lengths of follow-up** of patients, or in case-control studies, **is the time period** between the intervention and outcome **the same** for cases and controls?*		
18. *Were the **statistical tests** used to assess the main outcomes appropriate?*		
19. *Was **compliance with the intervention**/s reliable?*		
20. *Were the main **outcome measures used accurate** (valid and reliable)?*		
**Internal validity - confounding (selection bias)**<br>21. *Were the patients in different intervention groups (trials and cohort studies) or were the cases and controls (case-control studies) **recruited from the same population**?*		
22. *Were study subjects in different intervention groups (trials and cohort studies) or were the cases and controls (case-control studies) **recruited over the same period of time**?*		
23. *Were study subjects **randomised** to intervention groups?*		
25. *Was there adequate **adjustment for confounding** in the analyses from which the main findings were drawn?*		
26. *Were **losses of patients to follow-up** taken into account?*		
**Power**<br>27. *Did the study have **sufficient power** to detect a clinically important effect where the probability value for a difference being due to chance is less than 5%?*		
**TOTAL**		/23

Table S2: Realist terminology

CMO configuration	CMO configuring is a heuristic used in realist research to generate causative explanations pertaining to the data. The process draws out and reflects on the relationship of context, mechanism, and outcome of interest in a particular programme. A CMO configuration may pertain to either the whole programme or only certain aspects. Configuring CMOs is a basis for generating and/or refining the theory that becomes the final product of a realist review.
Conceptual Model	A conceptual model is a model made of the composition of concepts, which are used to help people know, understand, or simulate a subject the model represents.
Context	The term context has been used in this thesis to refer to conditions at different levels (micro, meso and macro) that impact on the operation of a programme or intervention.
Formal theory	Formal, or substantive, theory is existing theory within particular disciplines, such as sociology, economics, psychology, etc. Examples include game theory in economics and attachment theory in human development.
Mechanism	There are many definitions of mechanism, but a common thread is that they generate outcomes. In realist research, mechanisms are often considered to be underlying entities, processes, or structures which operate in particular contexts to generate outcomes of interest.
Middle-range theory	This is an implicit or explicit explanatory theory that can be used to assess programmes and interventions. "Middle-range" means that it can be tested with the observable data and is not abstract to the point of addressing larger social or cultural forces (i.e. grand theories).
Programme theory	This is theory about what a programme or intervention is expected to do, or how it is expected to work
Realism	Realism refers to a philosophy of science. It sits, broadly speaking, between positivism ('there is a real world which we can see and understand directly through observation') and constructivism ('we cannot know for sure what the nature of reality is, because all knowledge has been interpreted through human senses, language and culture').
Theory	There are multiple definitions of the word 'theory'. One simple way of thinking of theory is as an attempt to organise facts (proven or conjectural) into a structurally coherent system. Different types of theory are described in this glossary.

Table S3: Detailed summary of included studies

AUTHOR (YEAR)	LOCATION	AIM	STUDY DESIGN (QUALITY)	PARTICIPANTS (PRACTITIONERS)	PARTICIPANTS (PATIENTS)	MAIN OUTCOME(S)
Laws (2004)	**UK**	To collect national (anonymous) obesity data from primary care registers. • To develop treatment models for the management of obesity in primary care. • To facilitate the implementation of these treatment models into primary care. • To evaluate the impact of these models of care, and to inform future practice.	Quality improvement study<br><br>(Poor)	7 regions of UK – 10 practices in each region (except Aberdeen, where 20 practices recruited)	**N=1256**<br><br>**Mean BMI** was 36.9 kg m^2 (SD 5.4)<br><br>**Female = 74%**<br><br>**Mean age** = 50.6	91% received one of the core lifestyle interventions in the first 12 months.<br>34% achieved a clinical meaningful weight loss of 5% or more.
Lemay (2004)	New England, **USA**	To evaluate the effectiveness of BMI tables left in examination rooms as an intervention to encourage providers to calculate and record BMI scores in patients' medical records.	Prospective cohort study – intervention and control<br><br>(Fair)	Federally funded community health centre	**N=276**<br><br>No Mean BMI data<br><br>**Female = 75%**<br><br>**Mean age** = 39.2	Increased recording of BMI in patient's chart (49% vs 17%, p=0.0001)
Katz (2005)	**Israel**	The study objective was to determine if an interactive course would raise the self-efficacy of family practitioners (FPs) to treat obesity. The objectives of the course were to enrich the knowledge of FPs with	Pre-post test design; **no control** group; some qual interviews<br><br>(Poor)	Twenty-nine FPs (62% female) chose to participate in the course along with other Continuing Medical Education (CME)	No patient data	Self-reported increases in self-efficacy to treat obesity

AUTHOR (YEAR)	LOCATION	AIM	STUDY DESIGN (QUALITY)	PARTICIPANTS (PRACTITIONERS)	PARTICIPANTS (PATIENTS)	MAIN OUTCOME(S)
		up-to-date information on obesity and to raise their motivation to treat it.		courses. All participants work as FPs in public health care clinics throughout the country		
Flocke (2006)	Ohio, **USA**	The purpose of this study was to evaluate the effect of a practice-tailored intervention using Internet-based tools on the outcomes of clinician discussions of health behaviour change, referral to patient education and community resources, and patient movement in stage of motivation to change health behaviour.	A mixed-method longitudinal pre- and post-test study design.<br><br>(Good)	The seven participating practices included one solo physician practice, and single-specialty family practices ranging in size from 2 to 12 clinicians	**N=789**<br>Pre-intervention cohort: 368<br>Post-intervention cohort: 421<br><br>**Mean BMI =** 29.4 (SD 7.2; pre) / 29.5 (SD 7.7; post)<br>**Female = 77.5%** (pre) / 68.5% (post)<br><br>**Mean age = 42.5** (SD 14.1; pre)<br>/ 43.2 (SD 15.0; post)	Increased rates of discussion of diet (25.7% vs 20.2%), exercise (27.8% vs 16.9%), and **weight management (23.2% vs 16.3%, OR 1.57 (1.35-1.81), p<0.001).** Increased recommendation to consider looking into community programmes
Bordowitz (2007)	New York, **USA**	To evaluate if implementing an automatic calculation of BMI in an electronic medical record (EMR) vital signs section improves clinicians' documentation and treatment of overweight and obese patients.	Before – after study<br><br>(Poor)	10 attending physicians, 18 family medicine residents, and approximately 120 medical students who saw patients in the health centres.	**N=302** patient charts (153 charts before EMR and 149 charts after EMR).<br><br>**Mean BMI =** 29.2 (±6.6)(before); 28.5(±7.2)(after)	**Increased documentation of obesity from 31% to 71%** (prevalence ratio =2.30, 95% CI= 1.44–3.68)<br>Documentation of treatment of obese patients also improved, from 35% to 59%, (PR=1.84, 95% CI=1.19–2.86)

AUTHOR (YEAR)	LOCATION	AIM	STUDY DESIGN (QUALITY)	PARTICIPANTS (PRACTITIONERS)	PARTICIPANTS (PATIENTS)	MAIN OUTCOME(S)
					**Female = 66%** (before); 64% (after)<br><br>**Mean age** = 41(±16.1) (before); 36(±12.7) (after)	
Aspy (2008)	Oklahoma, **USA**	To test an implementation strategy that included audit with feedback, training, practice facilitation, and quality-circle meetings on screening and intervention rates for each of four behaviours: tobacco use, unhealthy diet, physical inactivity, and risky alcohol use.	Non-controlled, non-randomised intervention study<br><br>(Poor)	Of the 30 clinicians invited to participate, ten completed training and nine actually implemented changes in their process of care, resulting in an adoption rate of 30% (9/30)	No patient data	Increase in screening for diet (25.8% to 69.0%) and physical activity (0% to 23.6%)<br>Increase in brief intervention for diet (2.9% to 21.3%) and physical inactivity (2.9 to 21.0%)
Clark (2008)	Indiana, **USA**	To report the reach (in terms of primary care provider (PCP) prescriptions and patient first visits) of Take Charge Lite (TCL), a lifestyle weight management programme, in primary care.	Observational - audit<br><br>(Poor)	Two full-time and five part-time primary care providers (i.e., general internal medicine, family practice, and an adult nurse practitioner)	**N=2528**<br><br>**Mean BMI** = 38.2 (Range: 30-89; SD 7.8)<br><br>**Female = 71%**<br><br>No Mean age, just ranges	Increase in referral from 5% at baseline to around 20%<br><br>Of those screened positive for overweight/obesity, 5,034 (40.3%) received a TCL referral from their PCP
Ross (2008)	**UK**	To determine to what extent measures of success	QI – non-randomised	Of 65 practices agreeing to	**N=1906**	Mean weight change in those who attended and had data at

AUTHOR (YEAR)	LOCATION	AIM	STUDY DESIGN (QUALITY)	PARTICIPANTS (PRACTITIONERS)	PARTICIPANTS (PATIENTS)	MAIN OUTCOME(S)
		[in adult weight management] seen in intensive clinical trials can be achieved in routine primary care.	(Fair)	participate in the Counterweight Programme, 56 participated.	**Mean BMI =** 37.1 (6.0)<br><br>**Female = 77%**<br><br>**Mean age** = 49.4 (SD 13.5)	12 months (*n* = 642) was –3.0 kg (95% CI = –3.5 to –2.4 kg) and at 24 months (*n* = 357) was –2.3 kg (95% CI = –3.2 to –1.4 kg)
Ely (2008)	Kansas, **USA**	To conduct a pilot randomized trial of a chronic care model programme for obesity care in rural Kansas primary care.	Pilot RCT<br><br>(Good)	3 practices in 3 frontier rural counties	**N=107**<br>Active arm n=51.<br>Control arm n=56.<br><br>**Mean BMI** (SD) = 36 (7.3).<br><br>**Female = 71%**<br><br>**Mean age** = 49 (SD 14)	Day 180 mean (SD) weight change for the active and control arms, respectively, was −9.4 (10.3) pounds and −2.1 (10.7) pounds (*P* = 0.01 for difference)
Krist (2008)	Virginia, **USA**	To test the feasibility of an electronic linkage system (**eLinkS**) to help connect primary care practices with community resources to support behavioural counselling.	Non-randomized, pre–post design, feasibility evaluation<br><br>(Fair)	9 practices (Two sites were solo practices, five had 3 clinicians, one had 8 clinicians, and one (a family medicine residency programme) had 30 part-time clinicians and residents)	**N=5679**<br><br>No Mean BMI;<br>BMI ≥25–29 kg/m^2 = 1415 (25%)<br>BMI ≥30 kg/m^2 = 2197 (39%)<br><br>**Female = 64%**<br><br>**Median age** = 53 years	The % of patients with unhealthy behaviours who received intensive counselling through eLinkS (10%) exceeds practice norms. Advice given to 17% of obese patients – 12% of obese patients received a referral

AUTHOR (YEAR)	LOCATION	AIM	STUDY DESIGN (QUALITY)	PARTICIPANTS (PRACTITIONERS)	PARTICIPANTS (PATIENTS)	MAIN OUTCOME(S)
Schuster (2008)	Ohio, **USA**	To improve physician awareness and improve outcomes of overweight/obesity.	Before and after analysis of medical records<br><br>(Poor)	Family physicians who are part of Premier HealthNet group. Total of 100+ physicians in the group; 21 took part working in 5 offices.	**N=641**<br><br>**Mean BMI** = Intervention group at baseline (n=102): 29.6.<br><br>Enhanced intervention group (n=104): 30.66<br><br>**Female = 60%**<br><br>No Mean age;<br>32% >18-45<br>42% >46-65<br>26% >65	Increase in recording of obesity management in patient records: Intervention group: 2.4% to 9.2% (p=0.001).<br>Enhanced intervention group: 3.9% to 15.6% (p-0.002).<br>Increase in % physicians “comfortable” discussing obesity from 53% at baseline to 100% at 12 months (p=0.041).
Schriefer (2009)	North Carolina, **USA**	To evaluate whether or not the inclusion of a computerized BMI chart prompt as a vital sign on an electronic medical record would increase the likelihood that patients would receive a diagnosis of obesity and referral for treatment from their family physicians.	Before-and-after (non-randomised) controlled trial<br><br>(Good)	Family medicine residency programme clinic: 37 physicians in total (18 intervention and 19 control)	**N=846** (379 intervention and 467 control)<br><br>**Mean BMI** not reported<br>Prompt Control<br>Class I 46.7% 47.1%<br>Class II 29.3% 27.8%<br>Class III 24.0% 25.0%<br><br>**Female = 68%**<br><br>**No Mean age** data<br>20-30 10.3% 12.6%	Obese patients of physicians who had a BMI chart prompt in their medical records were significantly more likely than obese patients of physicians who did not receive a BMI chart prompt to receive a diagnosis of obesity (16.6% versus 10.7%; P=.016), and to receive a referral for diet treatment (14.0% versus 7.3%, P=.002) and exercise (12.1% versus 7.1%, P=.016).

AUTHOR (YEAR)	LOCATION	AIM	STUDY DESIGN (QUALITY)	PARTICIPANTS (PRACTITIONERS)	PARTICIPANTS (PATIENTS)	MAIN OUTCOME(S)
					31-40 19.5% 16.5%<br>41-50 21.9% 21.4%<br>>51 48.3% 49.5%	
Clark (2010)	Indiana, **USA**	To report on the percent of adult patients successfully screened for overweight or obesity, the percent of positive screens that received a primary care practitioner (PCP) referral, the percent of referred patients with Take Charge Lite (TCL) contact, the number of contacts, and the association between number of contacts and weight loss.	Retrospective evaluation<br><br>(Fair)	Five CHCs were used for the analyses reported in the paper. These were staffed with anywhere from six to 11 full- and part-time PCPs (i.e., general internal medicine, family practice, and nurse practitioners), as well as temporary internal medicine residents.	**N=12,487** eligible<br><br>**Mean BMI (SD).**<br>All TCL eligible: 34.7 (8.6)<br><br>**Female =** 71.2<br><br>**Mean age (SD)**<br>All TCL eligible: 43.3 (15.0)	Increase in referral from 5% at baseline to around 20%<br><br>Of those screened positive for overweight/obesity, 5,034 (40.3%) received a TCL referral from their PCP
Jay (2010)	New York, **USA**	To measure the impact of an obesity counselling curriculum on resident physicians' obesity counselling, operationalized as the use of 5As counselling strategies.	Non-randomized, wait-list/control design<br><br>(Good)	23 resident physicians in the primary care residency programme in this study	**Total N= 152**<br>n=74 (control) n=78 (intervention)<br><br>**Mean BMI** (SD) 34.5 (4.6)(control) 33.8 (3.8)(int)<br><br>**Female = 73%** (control); 71% (int)	Small but significant effects of the intervention on **quality of counselling** but not on the rate of counselling

AUTHOR (YEAR)	LOCATION	AIM	STUDY DESIGN (QUALITY)	PARTICIPANTS (PRACTITIONERS)	PARTICIPANTS (PATIENTS)	MAIN OUTCOME(S)
					**Mean age =** 43.5 (13.45) (control) 46.1 (13.7)(int)	
Wilson (2010)	Virginia, **USA**	To test a clinician-delivered intervention that utilized community resources for in-depth counselling for unhealthy behaviours including overweight. (**eLINKS)**	Pre/Post study design.<br><br>(Fair)	Nine primary care practices in a small town, semi-rural setting.	**N=146**<br><br>**Mean BMI**<br>Group counselling: 34.8.<br>Telephone counselling: 35.0.<br>Usual care: 31.0.<br><br>**Female = 70%**<br><br>**Mean age** (range)<br>Overall: 57 (23-90)	**Group counselling:** stat sig reductions in weight (3.5kg, p<0.001)<br>**Telephone counselling:** reduction in mean body weight (2.0kg, p=0.037)<br>**Usual care:** Small non-sig reductions in body weight (0.30kg)
Christian (2011)	Colorado, **USA**	To test the effect of a computerized support tool to enhance brief physician-delivered health lifestyle counselling to patients with increased metabolic risk factors during two usual care visits.	Prospective controlled trial<br><br>(Good)	Two large urban community-based health centres: the Pueblo Community Health Centres (PCHCs)	**N=263**<br>(130 control; 133 intervention)<br><br>**Mean BMI =** 33.8 (7.3)(control); 34.7 (7.4)(int)<br><br>**Female = 66.9%** (control); 69.9% (int)<br><br>**Mean age** = 50 (11.8)(control); 49.2 (13.0)(int)	Significantly more patients in the intervention group lost ≥5% of their body weight at 12 months than controls (26.3% vs 8.5%; odds ratio=3.86; *P*<0.01).

AUTHOR (YEAR)	LOCATION	AIM	STUDY DESIGN (QUALITY)	PARTICIPANTS (PRACTITIONERS)	PARTICIPANTS (PATIENTS)	MAIN OUTCOME(S)
Banerjee (2013)	Philadelphi a, **USA**	To determine how often obesity was included on the problem list and whether adding obesity to the problem list affected the rate at which it was addressed in future visits.	RCT<br><br>(Good)	Urban family medicine residency office. There were 51 providers seeing patients in this office: 39 resi-dents, nine faculty members, and three physician assistants.	**N=497**<br>Intervention: 258<br>Control: 239<br><br>**Mean BMI** =<br>Intervention: 34.9 (4.8)<br>Control: 34.3 (4.4)<br><br>**Female = 73%**<br><br>**Mean age =**<br>Intervention: 48.0 (16.9)<br>Control: 46.0 (16.4)	During the 5-month follow-up, obesity was addressed for 38 of 258 (14.7%) patients in the intervention group, compared with 11 of 239 (4.6%) patients in the control group (*P*<.001).
Jay (2013)	New York, **USA**	To assess whether a 5-h multimodal longitudinal obesity curriculum for residents on the basis of the 5As (assess, advise, agree, assist, and arrange) was associated with weight loss in their obese patients.	Retrospective chart review<br><br>(Good)	All 23 residents in the New York University School of Medicine (NYUSOM) primary care internal medicine residency programme (12 intervention; 11 control)	**N=87**<br>(intervention = 46; control = 41)<br><br>**Mean BMI =** 33.69<br><br>**Female = 72%**<br><br>**Mean age** = 48.26 (14.32)	**Mean Weight loss** of 1.53kg (SD 3.72) in intervention group compared to 0.30kg (SD 3.60) weight gain in control.<br>**Referrals:** 21 (45.7%) in intervention group versus 11 (26.8%) in control
Muo (2013)	New York, **USA**	To investigate the impact of the availability of height and weight data, in the form of BMI chart	Retrospective intervention study	Federally funded health centre.<br>30 residents and 14 attending	**N=406** (out of 486)<br><br>**Female = 71%**	**Significant increase** in the proportion of charts with **documented BMI** (2.5 vs 5%, P < 0.04).

AUTHOR (YEAR)	LOCATION	AIM	STUDY DESIGN (QUALITY)	PARTICIPANTS (PRACTITIONERS)	PARTICIPANTS (PATIENTS)	MAIN OUTCOME(S)
		reminders, on physician documentation of BMI and documentation of abnormal weight diagnosis and management.	(Fair)	internists provide primary care in the internal medicine practice.	**Mean age** = 54 (SD: 15)<br><br>No Mean BMI data:<br>**Number (%):**<br>Underweight (BMI<18.5) =**4** (0.8)<br>Normal weight (BMI>18.5 and <25.0) =**86** (17)<br>Overweight (BMI>25.0 and <30.0) =**152** (31)<br>Obese (BMI>30) =**218** (45)<br>Missing data for BMI calculation =**31** (6)	No difference in the rate of physician **documented weight-management plan** before and during the intervention (9.1 vs 9.8%, P = 0.75).
O'Grady (2013)	Minnesota, **USA**	To determine whether an automatic prompt for the clinician to recommend lifestyle changes to patients with a body mass index (BMI) >25 kg/m2 led to greater weight loss over a 3- to 6-month interval compared with the absence of a clinical reminder	Retrospective before-after case note review<br><br>(Fair)	No info on practitioners	**N=1600**<br>**Mean BMI** = 32.3 ± 7.4<br><br>**Female = 60%**<br><br>No Mean age:<br>18–29 = 281<br>30–44 = 562<br>45–59 = 1271<br>60–75 = 1086	The mean (± SD) change in weight was -0.51 (± 9.83) kg in the clinical reminder group, which did not significantly differ from the -0.35 (± 9.40) kg change in the controls (*P* = .64). Physician diagnoses of obesity or hyperlipidaemia were associated with weight loss, suggesting that formally noting these diagnoses contributes to successful weight loss

AUTHOR (YEAR)	LOCATION	AIM	STUDY DESIGN (QUALITY)	PARTICIPANTS (PRACTITIONERS)	PARTICIPANTS (PATIENTS)	MAIN OUTCOME(S)
Sinfield (2013)	**UK**	To investigate tailored implementation (i.e. investigating the context and barriers to change before selecting appropriate interventions) by two implementation groups as a part of a study to improve adherence to NICE guidelines on adult obesity in primary care.	Pilot study of small-group QI Approach<br><br>(Poor)	Implementation group A (n = 6) consisted of three medical practitioners, two PCT managers and a member of the research and development (R&D) support staff from a mental health trust. Implementation group B (n = 6) consisted of three PCT staff, two university academic staff and a member of the R&D support staff from a mental health trust.	No patient data	The practices had not identified as many people with obesity as predicted from population surveys (12% vs 26%) and interventions to assist weight loss were not delivered consistently, e.g. dietary advice was provided for approx. 39% of adults with obesity; referral to weight loss services was for <1% of patients.
Wilkes (2013)	Illinois, **USA**	To determine whether the Quality Improvement Collaborative (QIC) model can be feasibly implemented with limited resources at community	Qualitative evaluation<br><br>(Poor)	Five health centres, representing diverse settings across the Midwest,	No patient data	Qualitative evaluation. Participants reported improved ability to identify overweight patients in need of weight management. Three of the five teams reported an increasing

AUTHOR (YEAR)	LOCATION	AIM	STUDY DESIGN (QUALITY)	PARTICIPANTS (PRACTITIONERS)	PARTICIPANTS (PATIENTS)	MAIN OUTCOME(S)
		health centres in order to improve weight management programmes.		enrolled in the COACH collaborative		ability over time to engage their providers in order to increase referrals to the weight management programme.
Erickson (2014)	Minnesota, **USA**	(1) to evaluate the extent of guideline translation across organizations and (2) to assess the Omaha System as a method for translating system-level interventions and measuring outcomes.	Retrospective, mixed methods research<br><br>(Poor)	10 Administrators and 29 Clinicians (12 PHNs, five RNs, four NPs, two physicians, two physician assistants, and one each of the following: registered dietitian, physical therapist, occupational therapist, and physical therapy assistant.)	No patient data reported	On a scale of 1–5 (1 = low/neg; 5 = high/pos), the average Knowledge Behaviour Status (KBS) ratings across partner orgs increased over two points from baseline to 3 years follow-up.
Shungu (2015)	North Carolina, **USA**	To determine whether attaching a physical reminder card to patient encounter forms would increase electronic medical record (EMR) assessment of and documentation of obesity and dietary counselling	QI study – before and after<br><br>(Fair)	Data collected from one team, comprised of seven attending and 10 resident physicians, at a large urban academic family medicine practice	490 unique charts, of whom 211 (43.1%) were obese, were reviewed in the pre-intervention period. A total of 329 charts, of whom 127 (38.7%) were obese, were reviewed in the intervention period	Increase in assessment of obesity or morbid obesity, defined as clicking on obesity or morbid obesity as an active problem in the problem list in the EMR chart for the patient, **42.5% vs 28.0% (p=0.006)** but no difference in dietary counselling.

AUTHOR (YEAR)	LOCATION	AIM	STUDY DESIGN (QUALITY)	PARTICIPANTS (PRACTITIONERS)	PARTICIPANTS (PATIENTS)	MAIN OUTCOME(S)
Baer (2015)	Massachussetts, **USA**	(1) To develop EHR-based tools to help primary care providers identify, evaluate, and treat patients who are overweight or obese and (2) to conduct a cluster-randomized trial to examine the effectiveness of the new EHR-based tools.	Pragmatic clinical trial<br><br>(Good)	The 12 practices were divided into 23 clinical areas or teams	**Phase 2**<br>**N=20886 (control)**<br>**N=14779 (int)**<br>Adults with overweight/obesity<br><br>**Mean BMI** was 31.1 (control) and 31.2 (intervention).<br><br>**Female = 54% (control), 61% (int)**<br><br>**Mean age** = 53.3 years (control) and 53.7 (int)	Increase in recording of OV/OB on problem list, from 36% to 71%, compared to 16% to 8% in control group (p<0.0001). No significant differences in weight loss meds or nutrition counselling among pts with BMI>27
Steglitz (2015)	Illinois, **USA**	To examine whether implementation of an obesity intake protocol and an electronic health record (EHR) obesity management form could improve evidence based obesity practices and outcomes in a Federally Qualified Health Center (FQHC).	Before and after study design<br><br>(Fair)	12 clinicians recruited from a FQHC in West Chicago that served predominantly low-income, Hispanic adults	Pre (n=6624) Post (n=6960) *<br><br>**Mean BMI** not reported<br><br>Obese, n(%)<br>2237 (33.7)<br>2543 (36.5)*<br>Overweight<br>1499 (22.6)<br>1841 (26.4)*<br><br>**Mean age =** 41	**Clinician Surveys**<br>Clinicians self-reported that their practice of assessing physical activity, diet, and obesity-related medical conditions increased after the addition of the obesity intake protocol and weight mx form. Although their attitudes about treating obesity showed no significant change, clinicians also reported that the new protocol and EHR form made it easier to identify obese patients and increased their confidence

AUTHOR (YEAR)	LOCATION	AIM	STUDY DESIGN (QUALITY)	PARTICIPANTS (PRACTITIONERS)	PARTICIPANTS (PATIENTS)	MAIN OUTCOME(S)
						about managing obesity. **Exposure study (n=46 cases, 46 controls)** NS
Barnes (2015)	West Virginia, **USA**	To evaluate the impact of the Provider and Healthcare team Adherence to Treatment Guidelines (PHAT-G) intervention on adherence to current obesity clinical practice guidelines in an academic primary care centre.	Longitudinal practice-based evaluation project	PHAT-G was implemented in a primary care centre operated by the School of Medicine at a large state university in West Virginia, located approximately 75 miles from an urban area.	**N=100 records**<br><br>**Mean BMI** = 30.2<br><br>**No data reported on age or sex**	There was no routine documentation of BMI prior to the PHAT-G intervention. From time 1 (phase 1) to time 2 (phase 3), overall BMI documentation increased by 13%, which was significant (*P* < 0.01). Documentation rate of weight loss plan increased from 2 to 6 from time 1 to time 2 (NS)
Aveyard (2016)	**UK**	To establish whether physician brief intervention is acceptable and effective for reducing bodyweight in patients with obesity	parallel, two-arm, randomised trial<br><br>(Good)	137 primary care physicians at 57 practices from across the south of England	**N=1882**<br>Adults with obesity (BMI > 30 kg/m2) and a raised body fat percentage<br><br>**Mean BMI** was 34.9 (SD = 4.8).<br><br>**Female = 57%**<br><br>**Mean age** = 56 years (SD = 16.1)	As a result of the support intervention, 722 (77%) of 940 participants accepted referral to the weight management programme and 379 (40%) attended an appointment, compared with 82 (9%) participants who were allocated the advice intervention.
Goodfellow (2016)	**UK**	To determine whether a tailored implementation	Cluster randomised	16 control practices (6 rural,	**N=32079 (control)**<br>**N=17728 (int)**	There were no significant differences in the

AUTHOR (YEAR)	LOCATION	AIM	STUDY DESIGN (QUALITY)	PARTICIPANTS (PRACTITIONERS)	PARTICIPANTS (PATIENTS)	MAIN OUTCOME(S)
		intervention, in comparison with no intervention, increases the proportion of overweight/obese patients who are offered weight management as described in the NICE guidelines	trial<br><br>(Good)	10 urban); 12 intervention (3 rural, 9 urban)	Adults with overweight/obesity<br><br>**Mean BMI** was 30.2 (control) and 30.5 (intervention).<br><br>**Female = 52%**<br><br>**Mean age** = 50 years (control) and 53 (int)	proportion of patients offered a weight management programme between the control and intervention practices (15.1 % in the control practices, 13.2 % in the intervention practices, p=0.53).
Sturgiss (2017)	**Australia**	To describe the impact of participating in a pilot intervention for obesity management, The Change Programme, on the self-efficacy and confidence of Australian GPs	Nested mixed methods study<br><br>(Poor)	12 GPs, from 5 different general practices, 1 rural and 4 urban	No patient data reported	Increase in GPs' confidence and self-efficacy by providing them with a structured toolkit for the management of obesity.

Table S4: Studies broken down by intervention strategy

Author (year)	Participants	Description of intervention	Use of Theory	Main Outcome(s)
*Studies where **TRAINING** was main focus*				
Katz (2005)	29 doctors	Interactive, 12 clinical and psychological lectures, delivered by 'experts'.<br>2 lectures per session, followed by workshop and panel discussion (5pm – 9pm). Held monthly over 6 months.	Self-efficacy.<br>Social cognitive theory.<br>Transtheoretical model of behaviour change.	Self-reported **increases in self-efficacy** to treat obesity.
Jay (2010 and 2013)	23 resident physicians	5-h multimodal longitudinal obesity curriculum given over three weekly sessions.<br>Based on the 5As (assess, advise, agree, assist, and arrange) multiple active instructional methods including case studies, role-playing, standardized patients for counselling practice, and faculty-facilitated videotape review of residents counselling their own patients.	Skills such as behavioural assessment, goal setting, and motivational interviewing were stressed.<br>5As framework.	Small but significant effects of the intervention on **quality of counselling** but not on the rate of counselling.<br>**Mean Weight loss** of 1.53kg (SD 3.72) in intervention group compared to 0.30kg (SD 3.60) weight gain in control.<br>**Referrals:** 21 (45.7%) in intervention group versus 11 (26.8%) in control.
*Other studies that included **TRAINING** component*				
Laws (2004)<br><br>Ross (2008)	58 practices received training in intervention arm – all practice nurses	6–8 hour training programme for PNs.<br>Training manuals were provided to support formal workshops. Guidance was also provided on the use of Counterweight Programme patient education materials. A variety of teaching methods were used, including problem based learning through case studies, group discussion and practical exercises.	Adult learning theory	91% received one of the **core lifestyle interventions** in the first 12 months.<br>34% achieved a clinically meaningful **weight loss of 5%** or more of initial weight.
Aspy	10 clinicians	The training took place in a location convenient to all three	Stages of change.	**Increase in screening** for diet

Author (year)	Participants	Description of intervention	Use of Theory	Main Outcome(s)
(2008)		clinicians within a cluster, usually a hospital or clinician's office, and required about 2 hours for completion.<br>The motivational-interviewing workshop was conducted at the beginning of the project.<br>Five evidence-based training modules were developed by content experts in: motivational interviewing, weight loss, exercise, smoking cessation, and reduction of risky alcohol use. Each module included five components:<br>(1) a pre-test; (2) general information on the topic; (3) screening methods, and recommended brief and very brief interventions; (4) role-play scenarios; and (5) a post-test.	RE-AIM model.<br>Plan-do-study-act (PDSA) cycles.	(25.8% to 69.0%) and physical activity (0% to 23.6%).<br>**Increase in brief intervention** for diet (2.9% to 21.3%) and physical inactivity (2.9 to 21.0%).
Schuster (2008)	21 physicians took part, working in 5 offices	Few details provided: "focused academic detailing of the [national] guidelines, showing the physicians their own patient outcome data, and introducing minor systems innovations in the primary care office setting".<br>"Academic detailing is a successful tool for improving clinician outcomes. A peer, often with clinical skills and perceived as being academically credible, joins a small group of clinicians in a collegial and professional environment, reviews their clinical performance and advises them on mechanisms to improve their outcomes".	Academic detailing	**Increase in recording** of obesity management in patient records:<br>Intervention group: 2.4% to 9.2% (p=0.001).<br>Enhanced intervention group: 3.9% to 15.6% (p-0.002).<br>**Increase in % physicians "comfortable"** discussing obesity from 53% at baseline to 100% at 12 months (p=0.041).
Christian (2011)	2 community health centres 263 patients	3-hour training session.<br>Content included opportunities to reduce patient risk through lifestyle change, and how physicians would use these patient lifestyle change goal sheets to provide brief motivational interviewing counselling to help patients make changes in dietary and physical activity behaviours. Physician training also briefly covered the basics of the other behaviour change theories used to design the intervention.	The design of intervention feedback and content was based on four motivational theories: the Transtheoretical Model of Change, motivational interviewing, social-cognitive theory, and decision making.	**Significantly more** patients in the intervention group **lost ≥5%** of their body weight at 12 months than controls (26.3% vs 8.5%; odds ratio=3.86; *P*<0.01).
Wilkes	5 practices	Three in-person learning sessions took place in Chicago over the	Incorporated principles	Qualitative evaluation.

Author (year)	Participants	Description of intervention	Use of Theory	Main Outcome(s)
(2013)		course of two years.<br>Content included examples of Best Practices in weight management (e.g. Diabetes Prevention Programme); Review of Plan- Do- Study- Act (PDSA) Methodology; Motivational Interviewing; and Small Group Breakout Sessions to identify missing elements in current weight management programmes.	from Community Based Participatory Research (CBPR) methodology.	Participants reported **improved ability to identify** overweight patients in need of weight management. Three of the five teams reported an increasing ability over time to engage their providers in order to **increase referrals** to the weight management programme.
Erickson (2014)	10 administrators and 29 clinicians from 10 partner sites	A public health nurse (PHN) practice facilitator led partners in a learning collaborative utilizing face-to-face and web-based interactive trainings.<br>Partners learned about the obesity guideline (the Institute for Clinical Systems Improvement (ICSI) Prevention and Management of Obesity for Adults Guideline), organizational readiness to change, quality improvement strategies, adaptive leadership, patient-centred and patient-empowering conversational style and spirit (motivational interviewing), as well as how to develop an action plan with measurable aims.	5As framework and motivational interviewing.	On a scale of 1–5 (1 = low/neg; 5 = high/pos), the average Knowledge Behaviour Status (KBS) ratings across partner orgs **increased over two points** from baseline to 3 years follow-up.
Barnes (2015)	Staff at a large primary care centre	No detail provided on duration.<br>The intervention included education for both clinical support staff and primary care providers. The clinical support staff participated in a training session on the measurement, calculation and documentation of BMI. This training occurred with the support of the nurse manager and aimed to standardize the procedure for height and weight measurement, as well as calculation and documentation of BMI in the patient record. The educational component of phase 2 for providers was designed to target previously identified barriers to obesity management. Barriers and lack of knowledge related to treatment efficacy were addressed during the provider education session. Providers were	The theory of planned behaviour (TPB.	There was no routine documentation of BMI prior to the PHAT-G intervention. From time 1 (phase 1) to time 2 (phase 3), overall **BMI documentation increased** by 13%, which was significant ($P < 0.01$). **Documentation rate of weight loss plan** increased from 2 to 6 from time 1 to time 2 (NS).

Author (year)	Participants	Description of intervention	Use of Theory	Main Outcome(s)
		given the opportunity to discuss their frustration with previous attempts to manage obesity during the question and answer time following the education session.		
Steglitz (2015)	12 clinicians from a Federally Qualified Health Centre (FQHC).	No detail provided on duration.<br>A single training session that introduced clinic staff to the content of the new obesity management form, its location in the electronic health record (EHR), and details of the new protocol.	5As framework.	Clinicians self-reported that their practice of assessing physical activity, diet, and obesity-related medical conditions **increased** after the addition of the obesity intake protocol and weight mx form. Although their attitudes about treating obesity showed **no significant change**, clinicians also reported that the new protocol and EHR form made it **easier to identify obese patients** and **increased their confidence** about managing obesity.
Goodfellow (2016)	12 intervention practices (16 control)<br><br>17,728 intervention patients (32,079 control)	Group training to practice teams (GPs, practice nurses and health care assistants), including a presentation, discussion and provision of the resources (patient booklets, BMI charts, calories and portions leaflets, posters, information on referral pathways) was delivered by a registered dietitian.<br><br>The training lasted around 1 hour. Training began with a summary of the guidelines for professionals. Training addressed the issue of sensitively raising and discussing weight with patients. Training in waist measurement was provided with a live demonstration and explanation of the relationship of waist circumference to health risks. Training was given on how to	The authors did not draw on behavioural theory, relying instead on their own ideas on the strategies most suited to address the determinants, a process informed by the development of the Tailored implementation for chronic diseases (TICD) checklist.	Self-reported **increases in confidence, knowledge and skills** related to weight management, with respondents feeling better able to manage obese/overweight patients. However, there were **no significant differences** in the proportion of patients offered a weight management programme between the control and intervention

Author (year)	Participants	Description of intervention	Use of Theory	Main Outcome(s)
		assess patients' readiness to change their lifestyle and how to calculate energy requirements [418]. Professionals were also provided with example scripts to use in raising and discussing weight with patients. They were also given a prescriptive weight loss plan for patients because professionals felt that they did not always have sufficient knowledge or skill to advise patients on changes to diet.		practices (15.1% in the control practices, 13.2% in the intervention practices, p=0.53).
Aveyard (2016)	137 primary care physicians at 57 practices from across the south of England	Participating physicians received a 90 minute online course. The modules covered the rationale of the trial, the medical benefits of weight loss, and the mechanics of running the trial, but mostly consisted of filmed consultations with commentary to help physicians assimilate the skills necessary to deliver both interventions with confidence. The course also trained physicians to handle difficult situations that might arise in consultations and what to do in follow-up consultations. Fidelity was assessed by recording randomly selected consultations (i.e. consultations in which the randomisation card included a request to record). After each physician's session, the researcher listened to the recording and assessed whether key aspects of the intervention were delivered as intended. Feedback was provided to physicians where necessary to improve fidelity.	No formal theory cited, but the design of the intervention was informed by evidence that an offer of help to change is more motivating than advice to do so, by results of a trial of brief interventions for smoking cessation that showed that uptake is higher when the referral is enacted by the system rather than leaving patients to instigate it, and by evidence that external accountability is an important component of behavioural programmes; physicians were trained to ask the participant to return in 4 weeks to assess their progress.	As a result of the support intervention, 722 (77%) of 940 participants accepted referral to the weight management programme and 379 (40%) attended an appointment, compared with 82 (9%) participants who were allocated the advice intervention.

Author (year)	Participants	Description of intervention	Use of Theory	Main Outcome(s)
*Studies in which **TOOLS/RESOURCES** to **improve identification** of obesity were main strategy*				
Lemay (2004)	276 patients	The intervention consisted of posting prominent (2 feet by 3 feet), multi-coloured, laminated BMI tables in the exam rooms of one of the study site's three primary health care teams.	No formal theory cited, but authors suggest that inclusion of BMI calculations on the problem list of in progress notes may prompt provider to discuss weight management.	**Increased recording of BMI** in patient's chart (49% vs 17%, p=0.0001).
Bordowitz (2007)	10 attending physicians, 18 family medicine residents, and approximately 120 medical students who saw patients in the health centres.	In November 2003, an EMR was introduced in the two health centres. A feature of this particular EMR was an automatic calculation of BMI The BMI was automatically calculated when height and weight were entered and displayed in the vital signs section of the chart. The feature was not emphasized to providers, and there was no formal training about the BMI feature.	No formal theory cited, but authors cite studies showing that EMR reminders improve patient obtainment of preventive services such as screening and immunisations, and improve physician compliance with clinical guidelines.	**Increased documentation of obesity from 31% to 71%** (prevalence ratio =2.30, 95% CI= 1.44–3.68).<br>Documentation of treatment of obese patients also improved, from 35% to 59%, (PR=1.84, 95% CI=1.19–2.86).
Schriefer (2009)	37 physicians<br><br>846 patients	When a patient came for an office visit with a physician on an intervention group team, clinic staff obtained the patient's weight and height and computed the BMI from a calculation table that was provided by the researchers. The staff member then entered the height, weight, and BMI into the patient's electronic medical record. When the physician saw the patient, a computerised BMI appeared with other vital signs in the medical record.	USPSTF cited. No reference to formal theory, but the authors do cite evidence that chart prompts for physicians have proven to be effective for increasing the likelihood that physicians provide patients with preventive services, including immunizations and smoking cessation	Obese patients of physicians who had a BMI charts prompt in their medical records were **significantly more likely** than obese patients of physicians who did not receive a BMI chart prompt to receive a diagnosis of obesity (16.6% vs 10.7%; p=0.016), and to receive a referral for diet treatment (14.0% vs 7.3%; p=0.002) and

Author (year)	Participants	Description of intervention	Use of Theory	Main Outcome(s)
			services.	exercise (12.1% vs 7.1%; p=0.016).
Christian (2011)	2 community health centres<br><br>263 patients	Computer-based assessment of their motivational readiness to increase physical activity and make dietary changes just before a usual care visit. Then, computer's expert system generated a four- to five-page individualized, tailored report that provided feedback addressing participant-identified barriers to improving their physical activity and diet.	The design of intervention feedback and content was based on four motivational theories: the Transtheoretical Model of Change, motivational interviewing, social-cognitive theory, and decision making.	**Significantly more** patients in the intervention group **lost ≥5% of their body weight** at 12 months than controls (26.3% vs 8.5%; odds ratio=3.86; *P*<0.01).
Banerjee (2013)	51 providers (39 residents, 9 faculty members, and 3 physician assistants)<br><br>497 patients	The research team manually added obesity to the problem list of those 422 patients randomized to receive the intervention.	No formal theory cited. The USPSTF recommendations are referenced, as well as a study showing that physicians were more likely to address obesity with patients for whom the physician recorded obesity on the problem list than those patients who were identified as obese when they did not add obesity on the problem list during that visit.	During the 5-month follow-up, **obesity was addressed** for 38 of 258 (14.7%) patients in the intervention group, compared with 11 of 239 (4.6%) patients in the control group (*P*<.001).
Muo (2013)	30 residents and 14 internists<br><br>406 patients	A BMI chart reminder, which contained sections for weight, height and BMI, was stamped on all progress notes beginning on 1 July 2009. Following measurement of patients' height and weight at each visit, nursing staff completed height and weight sections of the BMI stamp. The BMI section was left blank to prompt physicians to calculate and record BMI. To acclimatise	No formal theory cited.	**Significant increase** in the proportion of charts with **documented BMI** (2.5 vs 5%, P < 0.04).<br>No difference in the rate of physician **documented**

Author (year)	Participants	Description of intervention	Use of Theory	Main Outcome(s)
		the providers to the location of BMI charts, coloured BMI charts were placed in conspicuous places in all examination rooms a few months before the implementation of the BMI chart reminders.		**weight-management plan** before and during the intervention (9.1 vs 9.8%, P = 0.75).
O'Grady (2013)	No info on practitioners<br><br>1600 patients	An automated clinical reminder for the clinician to recommend lifestyle modification for all adult patients with a BMI >25 kg/m^2 was added to the GDMS (Generic Disease Management System). A printed copy of the recommendations, including this reminder, was given to the patient.	No formal theory cited, though the authors state that automated clinical reminders are beneficial for improving screening uptake and have had some positive outcomes in diabetes and asthma management.	The mean (± SD) change in weight was -0.51 (± 9.83) kg in the clinical reminder group, which did **not significantly differ** from the -0.35 (± 9.40) kg change in the controls (*P = 0*.64).<br>Physician diagnoses of obesity or hyperlipidaemia were associated with weight loss, suggesting that formally noting these diagnoses contributes to successful weight loss.
Shungu (2015)	17 physicians (7 attending and 10 resident physicians)<br><br>490 patients	The intervention consisted of a brightly coloured, business-sized card being attached to the front of each billing encounter sheet by the MA prior to the encounter for all adult patients. Each reminder card included the following four questions: (1) What is your patient's BMI? (2) Did you document obesity? (3) Did you counsel on diet and exercise? and (4) Did you document counselling on diet and exercise?	No formal cited, but authors reference studies showing that documentation of obesity is important for primary care physicians for multiple reasons. Physicians are much more likely to address obesity if it is already recorded as a problem in the patient's chart.	**Increase in assessment of obesity** and morbid obesity, defined as clicking on obesity or morbid obesity as an active problem in the problem list in the EMR chart for the patient, 42.5% vs 28.0% (p=0.006) **but no difference in dietary counselling.**
Sturgiss	12 GPs, from 5	The programme consisted of a GP handbook, patient workbook	5As framework is cited.	**Increase in GPs' confidence and**

Author (year)	Participants	Description of intervention	Use of Theory	Main Outcome(s)
(2017)	different general practices, 1 rural and 4 urban<br><br>No patient data reported	and computer template. The GPs were not offered any training beyond the written handbook as in earlier qualitative work GPs stated they did not want a programme that required additional training.	Also, social cognitive theory.	**self-efficacy** (based on self-reported survey using a four-point Likert scale).
*Other studies in which **TOOLS/RESOURCES** to **improve identification** of obesity were used*				
Laws (2004)	58 practices<br><br>1256 patients	To prompt GP involvement, a desk-top flip chart was provided which included a range of tools to assist in patient screening and motivation.	The authors state that “The use of external stimuli to prompt changes in clinician behaviour has been shown to be effective and is consistent with learning theory”, without specifying any learning theories.	91% received one of the core lifestyle interventions in the first 12 months.<br>34% achieved a clinical meaningful weight loss of 5% or more.
Clark (2008)	7 primary care providers<br><br>2528 patients	Electronic review of medical records was used to determine age and body mass index (BMI) eligibility. BMI eligibility can be determined by 1) recorded BMI that may exist in the medical record from the most recent PCP visit, or 2) calculated BMI that used most recent weight and height to determine BMI. A positive screen (i.e. age greater than 17 and BMI 30 or more) resulted in an electronic eligibility reminder that PCPs saw at the time they were writing all other orders (prescription, referral, and procedure orders were carried out electronically). Reminders stated that some patients may not be appropriate for the programme, including pregnant women and those with serious	Cite USPSTF guidelines and 5As approach.<br><br>Also draws on the RE-AIM framework in terms of evaluating reach and considering implementation.	**Increase in referral** from 5% at baseline to around 20%<br>Of those screened positive for OW/obesity, 5,034 (40.3%) received a TCL referral from their PCP.

**Author (year)**	**Participants**	**Description of intervention**	**Use of Theory**	**Main Outcome(s)**
		mental illness.		
Krist (2008)	9 practices<br><br>5679 patients	Electronic linkage system - Utilizing the electronic medical record (EMR) as a platform, eLinkS was designed to (1) help clinicians systematically perform elements of the 5A's that are feasible in busy practice settings (i.e. asking about health behaviours, offering brief advice, and agreeing on next steps); (2) make it fast and easy to refer patients to intensive counselling outside the office; and (3) establish bidirectional communication between practices and community counsellors.	5As framework.<br><br>USPSTF guidelines are cited.	The % of patients with unhealthy behaviours who received intensive counselling through eLinkS (10%) exceeds practice norms. Advice given to 17% of obese patients – 12% of obese patients received a referral.
Ely (2008)	3 practices – no detail on practitioners	An electronic registry of obese patients was created with regular updates to physicians on patient motivation for weight loss, and obesity care recommendations for the next office visit. This registry information was in part derived from information collected during the telephone counselling sessions and included participant name, contact information, readiness to change regarding weight loss behaviour, weight loss attempts, methods employed in weight loss attempts, and facilitators and barriers to weight loss. The registry was updated monthly during the study.	Chronic Care Model.	Day 180 mean (SD) weight change for the active and control arms, respectively, was −9.4 (10.3) pounds and −2.1 (10.7) pounds (*P* = 0.01 for difference).
Erickson (2014)	10 Administrators and 29 clinicians from 10 partner sites<br><br>No patient data	Partner organizations incorporated system-level interventions such as the following into quarterly action plans:<br>• Relocation of scales to private locations and placement of working stadiometers conducive to work flow to facilitate body mass index (BMI) screening,<br>• Addition of prompts and reminders to the medical record system,<br>• Development of electronic tracking systems for panel or population management.	5As framework.<br><br>Also cites USPSTF and Institute for Clinical Systems Improvement (ICSI) Prevention and Management of Obesity for Adults Guideline.	On a scale of 1–5 (1 = low/neg; 5 = high/pos), the average Knowledge Behaviour Status (KBS) ratings across partner orgs **increased over two points** from baseline to 3 years follow-up.

Author (year)	Participants	Description of intervention	Use of Theory	Main Outcome(s)
Steglitz (2015)	12 clinicians<br><br>6960 patients	The nurse or medical assistant (MA) identified obese patients by entering height and weight data at intake. According to the new obesity management protocol, after rooming the patient, the nurse or MA helped him or her to complete an English or Spanish hardcopy version of a behaviour change goal checklist prior to the clinician's arrival. The clinician then discussed weight management with the patient while checking off on the EHR form the goals that the patient endorsed on the hardcopy version of the checklist. The endorsed goals auto-populated under the Health Goals section of the form and remained there to be reviewed in future visits.	5As framework.	Clinicians self-reported that their **practice of assessing** physical activity, diet, and obesity-related medical conditions **increased** after, as compared to before, the addition of the obesity intake protocol and weight management form. Although their attitudes about treating obesity showed **no significant change**, clinicians also reported that the new protocol and EHR form made it easier to identify obese patients and **increased their confidence** about managing obesity.
Baer (2015)	12 practices<br><br>14779 patients	**Reminders to measure height and weight**. Whenever a patient had no measurement of height in the EHR or no measurement of weight entered in the EHR within the past year, a reminder appeared on the summary screen, asking the provider to enter a height and/or weight for the patient. The EHR automatically calculated BMI from patients' most recent height and weight entries; therefore, any patient with both height and weight entered should have a BMI value in the EHR. **An alert asking providers whether they want to add overweight or obesity to the problem list**, for patients with BMI 25–29.9 or ≥30 kg/m^2, respectively. The alert appeared as a ''pop-up'' screen, and the provider had the option to add overweight or obesity or to dismiss the alert. This alert was added to an existing clinical alerting system, introduced in May 2010, which was	Several guidelines cited including USPSTF, National Institutes of Health (NIH), and American College of Physicians (ACP).<br><br>Also cite numerous studies showing under-identification of overweight and obesity in primary care.	**Increase in recording** of overweight/obesity on problem list, from 36% to 71%, compared to 16% to 8% in control group (p<0.0001). **No significant differences** in weight loss meds or nutrition counselling among pts with BMI>27.

Author (year)	Participants	Description of intervention	Use of Theory	Main Outcome(s)
		designed to improve the completeness of electronic problem list documentation for 17 other conditions.		
Barnes (2015)	1 primary care centre<br><br>100 patient records	**Reminder system:** A BMI chart was hung above each scale as a visual reminder for the nursing staff to measure, calculate and document BMI. Charts to calculate BMI were also available in the waiting room, nurses' stations and patient care rooms to increase the visual reminder effect on behaviour. One of the tools available was a treatment algorithm based on the clinical practice guideline recommendations. This treatment algorithm was displayed in patient care rooms as a reminder and a resource to both clinical staff and providers.	The theory of planned behaviour (TPB).	There was no routine documentation of BMI prior to the PHAT-G intervention. From time 1 (phase 1) to time 2 (phase 3), overall **BMI documentation increased** by 13%, which was significant ($P < 0.01$). Documentation rate of weight loss plan increased from 2 to 6 from time 1 to time 2 (NS).
Aveyard (2016)	137 primary care physicians at 57 practices from across the south of England	People who consented and were eligible to participate were handed a randomisation envelope to give to the general practitioner (GP), which included an appended record of the patient's height, weight, and BMI.	No formal theory cited, but the design of the intervention was informed by evidence that an offer of help to change is more motivating than advice to do so, by results of a trial of brief interventions for smoking cessation that showed that uptake is higher when the referral is enacted by the system rather than leaving patients to instigate it, and by evidence that external accountability is an important component of behavioural programmes;	As a result of the support intervention, 722 (77%) of 940 participants accepted referral to the weight management programme and 379 (40%) attended an appointment, compared with 82 (9%) participants who were allocated the advice intervention.

Author (year)	Participants	Description of intervention	Use of Theory	Main Outcome(s)
			physicians were trained to ask the participant to return in 4 weeks to assess their progress.	
Goodfellow (2016)	12 intervention practices (16 control)<br><br>17,728 intervention patients (32,079 control)	A poster and associated patient leaflet were provided to help professionals inform patients of the benefits of losing 5–10 % of their weight and to increase patient motivation through showing the benefits of a modest weight loss. Additional posters were also provided in paper and electronic format, including a poster to encourage patients to speak to a professional about their weight, plus BMI charts, and dietary guidance.<br>Posters for consulting rooms containing information on how to measure waist circumference were given as a visual reminder.	The authors did not draw on behavioural theory, relying instead on their own ideas on the strategies most suited to address the determinants, a process informed by the development of the TICD checklist.	Self-reported **increases in confidence, knowledge and skills** related to weight management, with respondents feeling better able to manage obese/overweight patients. However, there were **no significant differences** in the proportion of patients offered a weight management programme between the control and intervention practices (15.1% in the control practices, 13.2% in the intervention practices, p=0.53)
*Studies in which **TOOLS/RESOURCES** to improve **ease of referral** were main strategy*				
Clark (2008)<br><br>Clark (2010)	7 primary care providers<br><br>2528 patients<br><br>Five community health centres	Electronic review of medical records was used to determine age and body mass index (BMI) eligibility. BMI eligibility can be determined by 1) recorded BMI that may exist in the medical record from the most recent PCP visit, or 2) calculated BMI that uses most recent weight and height to determine BMI. A positive screen (i.e. age greater than 17 and BMI 30 or more) resulted in an electronic eligibility reminder that PCPs saw at the time they were writing all other orders (prescription, referral, and procedure orders are carried out electronically). Reminders	Cite USPSTF guidelines and 5As approach.<br><br>Also draws on the RE-AIM framework in terms of evaluating reach and considering implementation.	**Increase in referral** from 5% at baseline to around 20%<br>Of those screened positive for OW/obesity, 5,034 (40.3%) received a TCL referral from their PCP.

Author (year)	Participants	Description of intervention	Use of Theory	Main Outcome(s)
	12,487 patients	stated that some patients may not be appropriate for the programme, including pregnant women and those with serious mental illness. A single computer keystroke by the PCP led to the printing of a Take Charge Lite (TCL) prescription that was accompanied by a letter describing the free programme. The TCL prescription gave the telephone number to call to schedule a TCL appointment.		
Krist (2008)	9 practices<br><br>5679 patients	Electronic linkage system - Utilizing the electronic medical record (EMR) as a platform, eLinkS was designed to (1) help clinicians systematically perform elements of the 5A's that are feasible in busy practice settings (i.e., asking about health behaviours, offering brief advice, and agreeing on next steps); (2) make it fast and easy to refer patients to intensive counselling outside the office; and (3) establish bidirectional communication between practices and community counsellors.	5As framework .<br><br>USPSTF (2003) guidelines are cited.	The % of patients with unhealthy behaviours who received intensive counselling through eLinkS (10%) **exceeds practice norms**. Advice given to 17% of obese patients – 12% of obese patients received a referral.
Wilson (2010) – related to Krist (2008)	9 practices<br><br>146 patients	As above, but this paper focused on patients referred for weight loss. Patients could select from: group classes offered through a commercial weight loss programme (Weight Watchers); individual telephone weight loss counselling); computer- based counselling; or usual care, which consisted of any alternative the patient and clinician decided to pursue (e.g., counselling by the clinician or a decision not to address overweight).	5As framework.	**Group counselling**: stat sig reductions in weight (3.5kg, p<0.001)<br>**Telephone counselling:** reduction in mean body weight (2.0kg, p=0.037)<br>**Usual care:** Small non-sig reductions in body weight (0.30kg).
*Other studies in which **TOOLS/RESOURCES** to improve **ease of referral** were used*				

Author (year)	Participants	Description of intervention	Use of Theory	Main Outcome(s)
Flocke (2006)	7 practices	(1) A web-based health behaviour change resource including a database of community programmes and patient education materials, and (2) a health behaviour prescription pad.	5As framework.<br><br>Authors also cite evidence that clinicians are often unaware of community-based resources to which patients can be referred and lack mechanisms to efficiently direct patients to known resources.	**Increased rates of discussion** of diet (25.7% vs 20.2%), exercise (27.8% vs 16.9%), and **weight management (**23.2% vs 16.3%, OR 1.57 (1.35-1.81), p<0.001). **Increased recommendation** to consider looking into community programmes.
Ely (2008)	3 practices – no detail on practitioners	An electronic registry of obese patients was created with regular updates to physicians on patient motivation for weight loss, and obesity care recommendations for the next office visit. This registry information was in part derived from information collected during the telephone counselling sessions and included participant name, contact information, readiness to change regarding weight loss behaviour, weight loss attempts, methods employed in weight loss attempts, and facilitators and barriers to weight loss. The registry was updated monthly during the study. At the time of each registry update, physicians were sent feedback reports of patient progress and office visit recommendations.<br>**Decision Support.** Physicians were provided NIH obesity guidelines and regular updates based on electronic registry information. These updates included guideline-based obesity care recommendations and feedback on patient progress with weight loss behaviour change.	Chronic Care Model.	Day 180 mean (SD) weight change for the active and control arms, respectively, was −9.4 (10.3) pounds and −2.1 (10.7) pounds (*P* = 0.01 for difference).

Author (year)	Participants	Description of intervention	Use of Theory	Main Outcome(s)
Baer (2015)	12 practices<br><br>14779 patients	**Reminders with tailored management recommendations**, based on patients' BMI and other risk factors (e.g. hypertension, hyperlipidaemia, type 2 diabetes) included on the problem list or identified from medications or laboratory results. For each patient with BMI ≥25, one reminder appeared on the summary screen with a recommendation that was based on the NIH guidelines.<br>**A Weight Management screen with several features**, including tools to help providers assess patients' motivation to lose weight, calculate and set a 6-month weight loss goal, refer patients to other resources (e.g. nutritionist or medically monitored weight loss programme), and access more information.	Several guidelines cited including USPSTF, National Institutes of Health (NIH), and American College of Physicians (ACP).<br><br>Also cite numerous studies showing under-identification of overweight and obesity in primary care.	**Increase in recording** of overweight/obesity on problem list, from 36% to 71%, compared to 16% to 8% in control group (p<0.0001). **No significant differences** in weight loss meds or nutrition counselling among pts with BMI>27.
Goodfellow (2016)	12 intervention practices (16 control)<br><br>17,728 intervention patients (32,079 control)	At the time of the study, there were various community programmes to improve health and assist weight loss, some of which were available for patients to self-refer into, whilst others required a referral from a professional. Many professionals were not aware of the variety of services available or how to refer patients to them. During the intervention, professionals were asked to list all of the local services they were aware of. After visiting practices, the research team also searched for additional referral options, and then provided teams with a complete list of local services and referral pathways.	The authors did not draw on behavioural theory, relying instead on their own ideas on the strategies most suited to address the determinants, a process informed by the development of the TICD checklist.	Self-reported **increases in confidence, knowledge and skills** related to weight management, with respondents feeling better able to manage obese/overweight patients. However, there were **no significant differences** in the proportion of patients offered a weight management programme between the control and intervention practices (15.1% in the control practices, 13.2% in the intervention practices, p=0.53).

Author (year)	Participants	Description of intervention	Use of Theory	Main Outcome(s)
Aveyard (2016)	137 primary care physicians at 57 practices from across the south of England	Study staff ensured that patients who agreed to referral left the practice with an appointment.	No formal theory cited, but the design of the intervention was informed by evidence that an offer of help to change is more motivating than advice to do so, by results of a trial of brief interventions for smoking cessation that showed that uptake is higher when the referral is enacted by the system rather than leaving patients to instigate it, and by evidence that external accountability is an important component of behavioural programmes; physicians were trained to ask the participant to return in 4 weeks to assess their progress.	As a result of the support intervention, 722 (77%) of 940 participants accepted referral to the weight management programme and 379 (40%) attended an appointment, compared with 82 (9%) participants who were allocated the advice intervention.
*Studies in which **AUDIT/FEEDBACK** was main strategy*				
Aspy (2008)	10 clinicians	**Facilitation**<br>One practice-enhancement assistant was assigned to each geographic cluster of clinicians. Each practice-enhancement assistant worked with three clinician–office staff teams in a single geographic cluster to implement the new screening and intervention methods using PDSA quality-improvement cycles, a	Stages of change.<br>RE-AIM model.<br>Plan-do-study-act (PDSA) cycles.	**Increase in screening** for diet (25.8% to 69.0%) and physical activity (0% to 23.6%)<br>**Increase in brief intervention** for diet (2.9% to 21.3%) and physical inactivity (2.9 to

Author (year)	Participants	Description of intervention	Use of Theory	Main Outcome(s)
		skill they had developed in prior projects. The practice-enhancement assistants also performed monthly chart audits to provide feedback to the clinicians on their progress. To ensure data accuracy, all practice-enhancement assistants audited the same set of training charts and discussed any differences until agreement was 100%. The practice-enhancement assistants worked closely with the nurses and medical assistants to modify office routines, forms, and computer templates; they helped each team identify community resources; and they helped the team find or develop patient education materials.		21.0%).
*Other studies in which **AUDIT/FEEDBACK** was used*				
Laws (2004)<br><br>Ross (2008)	58 practices<br>1256 patients	A 1 h workshop was conducted with GPs and PNs in each practice to feedback the audit results, to discuss the treatment pathway and to set priorities for implementation. By highlighting the burden of obesity in each practice and the current levels of screening and intervention, it was hoped that GPs would support the implementation of a more consistent and structured approach to weight management in the practice. The main role of the GP was to identify suitable patients for weight management intervention during routine clinical practice and to refer on to the PNs. This involved raising weight as an issue with appropriate patients and possibly discussing the benefits of a 5–10% weight loss.	Adult learning theory.<br><br>The authors also cite evidence that feedback of audit results related to current clinical practice can help to change future practice.	91% received one of the core lifestyle interventions in the first 12 months.<br>34% achieved a clinically meaningful weight loss of 5% or more of initial weight.
Ely (2008)	3 practices – no detail on practitioners	An electronic registry of obese patients was created with regular updates to physicians on patient motivation for weight loss, and obesity care recommendations for the next office visit. This registry information was in part derived from information collected during the telephone counselling sessions and included participant name, contact information, readiness to change	Chronic Care Model.	Day 180 mean (SD) weight change for the active and control arms, respectively, was −9.4 (10.3) pounds and −2.1 (10.7) pounds (P = 0.01 for difference).

Author (year)	Participants	Description of intervention	Use of Theory	Main Outcome(s)
		regarding weight loss behaviour, weight loss attempts, methods employed in weight loss attempts, and facilitators and barriers to weight loss. The registry was updated monthly during the study. At the time of each registry update, physicians were sent feedback reports of patient progress and office visit recommendations.		
Schuster (2008)	21 physicians<br><br>641 patients	Physicians presented with information (feedback) on outcomes.	No formal theory cited.	**Increase in recording** of obesity management in patient records: Intervention group: 2.4% to 9.2% (p=0.001).Enhanced intervention group: 3.9% to 15.6% (p-0.002).<br>**Increase in % physicians "comfortable"** discussing obesity from 53% at baseline to 100% at 12 months (p=0.041).
Wilkes (2013)	5 health centres<br><br>No patient data	Teams learned to implement the Quality Improvement (QI) model, which instituted rapid cycles of change (Plan- Do- Study-Act) framework. To track health centre QI project implementation and facilitate peer- learning across sites, a password-protected website was developed and refined based on participant feedback. Teams were asked to enter monthly updates on the website to document their experience and progress implementing rapid cycle QI within their weight management programmes. Monthly conference calls facilitated sharing experience across practice sites regarding QI implementation, challenges and successes.	QI approaches, e.g. PDSA cycles.	Participants reported **improved ability to identify overweight patients** in need of weight management. Three of the five teams reported an **increasing ability over time to engage their providers** in order to increase referrals to the weight management programme.
Barnes (2015)	1 primary care centre<br><br>100 patient	Once every week, the project director communicated with the clinical staff and providers regarding the project objectives. This reminder communication occurred through a card in their clinic mailbox. Audit and feedback results were communicated as part	The theory of planned behaviour (TPB).	There was no routine documentation of BMI prior to the PHAT-G intervention. From time 1 (phase 1) to time 2

Author (year)	Participants	Description of intervention	Use of Theory	Main Outcome(s)
	records	of the reminders via email at the mid-point of the 6-week implementation phase.		(phase 3), overall **BMI documentation increased** by 13%, which was significant (*P* < 0.01). Documentation rate of weight loss plan increased from 2 to 6 from time 1 to time 2 (NS).
Erickson (2014)	29 clinicians and 10 administrators and from 10 partner sites<br><br>No patient data	The consultants held conference calls and webinars to provide follow-up and support, and to conduct evaluation.<br>Following the initial 12-month intervention, the PHN practice facilitator met quarterly with each partner organization to assess action plan progress and barriers to success, and to offer guidance and resources supportive of guideline implementation.<br>The PHN practice facilitator applied the 5As framework to her system-level facilitation activities.	5As framework and motivational interviewing.	On a scale of 1–5 (1 = low/neg; 5 = high/pos), the **average Knowledge Behaviour Status (KBS) ratings** across partner orgs **increased** over two points from baseline to 3 years follow-up.
*Studies in which **NETWORKS/QUALITY CIRCLES** were main strategy*				
Sinfield (2013)	Two implementation groups with 12 people in each (6 clinicians)	Two facilitated implementation groups explored tailoring to improve adherence to NICE guidelines on adult obesity in primary care.<br>Tailoring involved two key steps. The first involved investigation of context and the prevailing barriers to change in which a variety of methods may be used with professionals and patients including: interviews, focus groups, questionnaires and observation. The second step involved the selection of intervention methods chosen to account for the barriers identified.	No formal theory, but literature on implementation barriers cited. A systematic review of randomised controlled trials found that tailored interventions were more effective than no intervention or to dissemination of guidelines and educational materials alone.	Identification of barriers and facilitators to weight management in primary care.

Author (year)	Participants	Description of intervention	Use of Theory	Main Outcome(s)
*Other studies in which **NETWORKS/QUALITY CIRCLES** were used*				
Laws (2004)<br><br>Ross (2008)	56 practices	Weight management advisers, all registered dietitians with specialist postgraduate training and experience in obesity management, led and facilitated implementation of the programme. They provided protocols and training materials for practice staff and patient education materials. Peer support in the practices was provided once or twice each month by the weight management adviser until practice nurses achieved competency and confidence. Mentoring usually took 6 months. Subsequent visits by weight management advisers were to assist with auditing outcomes, provision of materials (for example, information leaflets), and training of new staff members.	Adult learning theory.	Mean weight change in those who attended and had data at 12 months (n = 642) was –3.0 kg (95% CI = –3.5 to –2.4 kg) and at 24 months (n = 357) was –2.3 kg (95% CI = –3.2 to –1.4 kg).
Aspy (2008)	10 clinicians	Quality Circle: During each cycle, the three clinician teams in each cluster met three times (at 2, 4, and 6 months) with their practice enhancement assistant and the principal investigator to review progress and share ideas. Performance data were shared, and specific techniques were described and discussed. The clinician, a nurse or medical assistant, and an office manager from each practice generally participated in these meetings.	Stages of change.<br>RE-AIM model.<br>Plan-do-study-act (PDSA) cycles.	**Increase in screening** for diet (25.8% to 69.0%) and physical activity (0% to 23.6%)<br>**Increase in brief intervention** for diet (2.9% to 21.3%) and physical inactivity (2.9 to 21.0%).
Wilkes (2013)	5 health centres<br><br>No patient data	Combating Obesity at Community Health Centres (COACH) Quality improvement collaborative – learning sessions (PDSA methods and one based on Diabetes Prevention Programme); website for evaluation; conference calls for knowledge sharing. Supported by financial resources and evidence-based tools to implement this QIC.	QI approaches, e.g. PDSA cycles.	Participants reported improved ability to identify overweight patients in need of weight management. Three of the five teams reported an increasing ability over time to engage their providers in order to increase referrals to the weight management programme.

NS= Not significant, PHN = Public Health Nurse

Table S5: If-Then-Because statements

**1. *Discussion of weight***			
**Level**	**If**	**Then**	**Because**
Individual (patient and practitioner)	Patients have weight-related co-morbidities	Discussion of weight is more likely	Patients and practitioners may feel it is a legitimate (medical) reason to talk about their weight
Individual (practitioner)	Practitioners receive training on the health risks associated with obesity	Discussion of weight is more likely	Practitioners have more knowledge of the risks
	Practitioners receive training on how to raise the issue of weight sensitively	Discussion of weight is more likely	Practitioners feel more confident in raising the issue of weight
Interpersonal	Practitioners are challenged to think about size/weight discrimination	Discussion of weight is more likely to be done in a supportive, non-judgmental way	Practitioners have reflected on their own attitudes and assumptions
	Practitioners are overweight/obese themselves	Discussion of weight is more likely	Practitioners feel they can empathise more effectively with patients
Institutional	Practitioners feel they have the support of other practice staff	Discussion of weight is more likely	Weight management is valued as a priority
Infrastructural	Practitioners are reimbursed for preventive health care	Discussion of weight is more likely	There is a financial incentive
**2. *Recording of weight***			
**Level**	**If**	**Then**	**Because**
Individual (patient)	Patients want support with weight loss	Recording of weight/BMI is more likely	There is motivation for weight monitoring
Individual (practitioner)	Scales are present in all consulting rooms	Recording of weight/BMI is more likely	There is increased awareness
Interpersonal	BMI charts are visible to patients in waiting room or consulting room	Recording of weight/BMI is more likely	Patients and practitioners may feel more able to raise the issue of weight
Institutional	The process for recording BMI is automated	Recording of BMI is more likely	It is quick and easy
Infrastructural	There are systems in place for monitoring obesity	Recording of weight/BMI is more	Practices may value the purpose of

	trends and comparing trends across practices	likely	recording for audit/QI purposes
		3. Referral to WMS	
**Level**	**If**	**Then**	**Because**
Individual (patient)	Patients with obesity have weight-related co-morbidities	Referral to WMS is more likely	Their problem can be medicalised and practitioners feel more comfortable discussing it
	Patients and practitioners are both female	Referral to WMS is more likely	There is an expectation that they are more likely to attend
	Patients are not socio-economically deprived	Referral to WMS is more likely	Practitioners may believe that they are more likely to attend
Individual (practitioner)	Practitioners are familiar with the options available	Referral to WMS is more likely	Practitioners have increased awareness and familiarity
Interpersonal	GPs appear confident and optimistic about the service	Referral to WMS is more likely	Patients are more likely to trust the referral
Institutional	Practitioners have been shown evidence of effectiveness of WMS	Referral to WMS is more likely	Practitioners have increased confidence in service
	The recording of BMI is routine within the practice	Referral to WMS is more likely	Practitioners are aware of the diagnosis of obesity
	WMS have made efforts to improve communication with practices	Referral to WMS is more likely	Practitioners have increased trust in service
	There is an obesity lead/champion within the practice	Referral to WMS is more likely	Weight management is given priority and visibility within the practice
	The referral process is fast and easy to use	Referral to WMS is more likely	It becomes automatic and habitual for the practitioner
Infrastructural	There is a high prevalence of obesity in the area	Referral to WMS is more likely	Practitioners recognise the need for support because obesity is seen as a local priority
	There is a high prevalence of obesity in the area	Referral to WMS is less likely	Overweight is normalised and not seen as a priority

Table S6: Contextual factors with illustrative examples

LEVEL	Contextual factor	Illustrative example/quote
**Micro (individual/interpersonal)**	Patient characteristics (e.g. Age, Gender, Ethnicity, SES, BMI, Co-morbidities)	*"eLinkS was used more for middle-aged patients and women, perhaps because the available services (e.g., Weight Watchers) appealed to this group." (Krist, 2008)*
	Practitioner characteristics (e.g. Experience, BMI)	To explain why the automatic BMI calculation was more successful for improving documentation and treatment of adults with obesity than it was for those who were overweight:<br>*"One possible reason for this discrepancy is that physicians may check BMI only when the patient is noticeably obese. Another reason may be that physicians check BMI only when the patient has a comorbid illness."* (Bordowitz, 2007)
	Stigma / Fear of causing offence	*"In some practices patients may view their problem list, either on a visit summary or through an online portal. If obesity is added to the problem list, the patient could become aware of this. While this may have a positive outcome in patient motivation to lose weight or desire to discuss obesity with the provider, it could also have unintended, negative psychological or social consequences."* (Bannerjee)
**Meso (institutional)**	Competing priorities / Presence of "alert fatigue"	*With the influx of a myriad of electronic reminders, clinicians may have simply succumbed to "alert fatigue" and ignored this reminder. (O'Grady)*
	Inflexibility of EHRs	*For this particular project, electronic health records were both a blessing and a curse. Certainly it was helpful to be able to codify the desired behaviours in an EMR template. However, most of the EMRs were so inflexible that it proved difficult to insert the screening questions within the record's vital-signs section* (Aspy)
	Practice culture	*General practice is under great pressure consequent upon the ageing population and growing levels of multimorbidity. Primary care teams may find themselves having to prioritise their activities and may be too busy*

		caring for those who, for example, already have type 2 diabetes to be able to devote much time to people who are overweight or obese. (Goodfellow)
**Macro (infrastructural)**	High prevalence of obesity, leading to normalisation	*Given the high prevalence of overweight and obesity in the study population, physicians and patients alike may have a high threshold for detecting overweight and obesity.* (Muo, 2013)
	Timing of external events	*For example, one practice's members cited a time-intensive but financially advantageous research collaboration that started simultaneously with this study's intervention as a reason few practice members used the Resource during the study period.* (Flocke)<br><br>*An additional factor may have been the publication of an update of the NICE obesity guidelines during the course of the study. However, the new guideline did not make substantive changes to the recommendations for primary health care teams* (Goodfellow)
	Funding for weight management	*Family physicians may have little motivation or incentive to bring the issue of obesity to the attention of the patient because physicians are not reimbursed by insurance companies for diagnosing obesity* (Schriefer)
